# Source Characterization of Multiple Reactive Species at an Abandoned Mine Site Using a Groundwater Numerical Simulation Model and Optimization Models

**DOI:** 10.3390/ijerph18094776

**Published:** 2021-04-29

**Authors:** Michael Saah Hayford, Bithin Datta

**Affiliations:** College of Science and Engineering, James Cook University, Townsville, QLD 4814, Australia

**Keywords:** optimal contaminant source characterization, multiple species reactive transport, contaminated aquifer, evolutionary optimization, linked simulation optimization

## Abstract

The most important first step in the management and remediation of contaminated groundwater aquifers is unknown contaminant source characterization. Often, the hydrogeological field data available for accurate source characterization are very sparse. In addition, hydrogeological and geochemical parameter estimates and field measurements are uncertain. Particularly in complex contaminated sites such as abandoned mine sites, the geochemical processes are very complex and identifying the sources of contamination in terms of location, magnitude, and duration, and determination of the pathways of pollution become very difficult. The reactive nature of the contaminant species makes the geochemical transport process very difficult to model and predict. Additionally, the source identification inverse problem is often non-unique and ill posed. This study is about developing and demonstrating a source characterization methodology for a complex contaminated aquifer with multiple reactive species. This study presents linked simulation optimization-based methodologies for characterization of unknown groundwater pollution source characteristics, i.e., location, magnitude and duration or timing. Optimization models are solved using an adaptive simulated annealing (ASA) optimization algorithm. The performance of the developed methodology is evaluated for different complex scenarios of groundwater pollution such as distributed mine waste dumps with reactive chemical species. The method is also applied to a real-life contaminated aquifer to demonstrate the potential applicability and optimal characterization results. The illustrative example site is a mine site in Northern Australia that is no longer active.

## 1. Introduction

The remediation of contaminated aquifers is a challenging task in groundwater resource management. Effective and reliable management of groundwater resources first requires the identification of the contaminant sources [1]. Numerous problem-solving approaches have been proposed in recent decades to address contaminant source identification problems [2,3]. Among these approaches, linked simulation-optimization models have been progressively applied to identify groundwater contaminant source characteristics in contaminated aquifers. However, over the years, most researchers studied point sources or ideally shaped non-point pollution sources [4,5,6]. More so, such studies considered the contaminants in the transport model as non-reactive in homogeneous geological media while solving the unknown source identification optimization problem. Even though such models can be simulated by considering ideally shaped or point sources, they cannot be used to determine the characteristics of distributed groundwater contaminant sources with reactive contaminants [7,8]. Furthermore, in highly heterogeneous geological media involving geochemical reactions (both kinetic and equilibrium reactions) of reactive contaminant species, the simulation of the transport process becomes more complex and difficult [9]. The main aim of this study was to show the feasibility and applicability of an integrated simulation and optimization approach for unknown contamination source identification in a regional-scale contaminated aquifer with very complex hydrogeological and geochemical processes. The aim was also to demonstrate how a very limited amount of field measurements, a typical scenario in many such field problems, can be utilized for developing both flow and reactive transport models for a complex contaminated aquifer. The methodology and the performance evaluation results presented here demonstrate the feasibility of solving the very complex unknown groundwater pollution sources characterization in terms of intensity, location, and duration with limited field measurements, while successfully integrating the uncertain and complex geochemical environment in a mine site.

The optimal characterization of the contaminant sources requires accurate simulation of the flow and transport processes occurring in the contaminated aquifer. The contamination scenario at an operational or abandoned mine site is generally very complex due to geological heterogeneity, distributed sources and the presence of multiple reactive species in the mined mineral ores [10]. In addition, at abandoned mine sites, monitoring of spatially- and temporarily-varying hydraulic heads and concentrations is usually very sparse and inadequate. Optimal characterization of contaminant sources also requires the use of an accurate flow and transport simulation model [11,12,13,14,15]. Accurate and reliable identification of contaminant sources at an abandoned mine site is, therefore, especially complex and difficult. Source characterization is the first step towards reliable and sustainable contamination remediation. The issue of accurately characterizing contaminant sources at poorly monitored abandoned mining sites is crucial since the soil matrix and groundwater contamination have important influences on human health, vegetation and ecological systems [16]. Thus, solving contaminant source problems in complex aquifers characterized by many aquifer parameter dissimilarities requires methods that are robust, efficient and able to handle data uncertainty [17].

To optimally identify spatially-distributed groundwater contamination sources, the source flux, activity duration, and time of initiation need to be determined [18]. However, when multiple reactive chemical species are present as contaminants, identification of the sources in terms of the individual species involved is required before developing a remediation strategy. 

Previous distributed contamination source characterization models for typical mine sites were reported in Jha and Datta [19]. They developed a linked simulation optimization-based methodology for estimating the release histories of spatially-distributed fixed pollution sources at an illustrative abandoned mine site, but the pollution sources were considered as conservative. In their method, adaptive simulated annealing (ASA) was used as an optimization algorithm to determine source concentrations at the pit. Similarly, Ayvaz [20] developed a genetic algorithm-based simulation-optimization model to determine the spatial distributions and source fluxes of areal groundwater pollution sources. This model was evaluated on a simple hypothetical aquifer model under ideal conditions. Eshafani and Datta [21] developed genetic programming models as surrogate models for the characterization of distributed contaminant sources at a contaminated mine site. Genetic programming-based trained surrogate models were used to approximate complex transport processes involving reactive species. However, this study did not individually characterize the constituents of multiple contaminant species. 

The objective of the present research was to develop and illustrate the use of a linked simulation-optimization approach to solving distributed groundwater pollution source identification problems in a complex groundwater system, with emphasis on multiple reactive species. To the best of our knowledge, the potential of this approach for addressing multiple reactive species contaminant sources is still largely unexplored. 

For this work, it was assumed that the observed concentrations are measured at several monitoring locations, dispersed in space, and monitoring contaminant transients over a period. If there are multiple contamination sources in an aquifer, each monitoring location detects a mixture of contamination fields (plumes) originating from different locations. It is assumed that each contaminant source releases a different geochemical constituent that is mixed in the aquifer, and that the resultant mixture is detected at the observation locations. Additionally, the geochemical constituents are reactive, and their transport is impacted by geochemical reactions or other fluid/solid interactions in the porous media where the flow occurs. The aim is to identify the number, locations and activity times of contamination sources.

The scenario of having very limited flow and concentration measurement data was incorporated to represent the typical situations of contaminated sites. The effective integration of a source identification optimization modelling technique with an accurate simulation of contaminant transport from distributed sources with complex pollutant geochemistry is addressed. The proposed approach is then evaluated for efficiency, accuracy and applicability. In the proposed approach, the groundwater flow and reactive transport processes are simulated by modelling the aquifer system of an abandoned (no longer functioning) uranium mine located in the Northern Territory of Australia. The three-dimensional coupled physical and chemical transport process simulator HYDROGEOCHEM 5.0 [22] is used to numerically simulate the flow and reactive chemical transport processes. 

The objectives of our study are three-fold. The first objective is to evaluate the performance of an inverse source identification problem formulation to identify contaminant source characteristics based on a synthetic case study with highly complex hydrogeological and aquifer properties. This is important because, despite the advantages of using a simulation-optimization formulation to solve an inverse unknown groundwater problem, this approach has largely been applied to statistical-type heterogeneities, knowing well how the type of heterogeneities largely influence mass transport. 

The second objective is to test the efficiency and advantages of using an adaptive simulated annealing optimization algorithm (ASA) to demonstrate the feasibility of characterizing multiple species concentrations from distributed sources. ASA is a global optimization algorithm that depends on randomly sampling important parameter space [23]. In contrast to the deterministic approaches, the exponential annealing schedules in the ASA allow resources to be used adaptively on re-annealing and convergence in all dimensions, guaranteeing extensive global search in the first phases of the search and quick convergence in the final phases of the projected stopping criteria in the formulated problem of source identification [24]. While ASA optimization has been applied to a variety of optimization problems [25], this study applied ASA to characterize multiple contaminant species in source identification problem.

The third objective is to provide an optimization benchmark case that allows optimization strategies on objective functions defined over a discrete domain and inspired by real applications. The performance of the developed method is evaluated using field measurement data in a real complex contaminated aquifer system. It initially uses synthetic data (to be able to evaluate the performance for different variations of the scenarios in the field) on the multiple species concentration identification at the distributed sources. The reason being, actual contaminant source characteristics are unknown. Therefore, such a methodology can be validated using synthetic simulated data only. The geophysical aquifer characteristics are based on field conditions. The performance evaluation is carried out using limited concentration data obtained at the study site to characterize the contaminant sources. The developed method is applied to an abandoned uranium mining site in the Northern Territory, Australia, and an associated contaminated groundwater system.

## 2. Materials and Methods

### 2.1. Study Area

The Rum Jungle Mine site is located in the tropical wet-dry climatic region of northern Australia. The mine areas consist of the east branch of the Finniss River approximately 8.5 km upstream of its confluence with the west branch of the Finniss River. The region is characterized by a tropical savannah-like climate and typically receives approximately 1500 mm of annual rainfall. Most of this rainfall (90% or more) occurs during a wet season that lasts from November to April, with no sustained rainfall occurring from May to September. Surface water enters the mine site from the east via the upper east branch of the Finniss River and from the southeast via Fitch Creek. River flows vary in response to intra annual variability in rainfall and changes over the course of a year [26]. The mine area mineral field contains polymetallic ore deposits, such as the Ranger and Woodcutters ore deposits. The mine site includes the Giant’s reef fault. The main lithological units are the Rum Jungle Complex and meta-sedimentary and subordinate meta-volcanic rocks of the Mount Partridge Group. The Rum Jungle Complex consists mainly of granites and the Mount Partridge Group consists of sedimentary units: Geolsec formation, the Whites formation, the Coomalie Dolostone, and the Crater formation [27].

#### Methods

In this section, a general mathematical formulation of the source identification problem and its notation are presented. The proposed linked simulation-optimization method of source characterization has a two-phase structure of numerical simulation and optimization.

### 2.2. Groundwater Flow and Transport Simulation

#### 2.2.1. Modelling of Groundwater Flow and Contaminant Transport

The 3D finite element-based reactive transport simulator HYDROGEOCHEM 5.0 was utilized in this study to model the aquifer flow and transport processes. The flow and reactive transport models are described in the following sections.

#### 2.2.2. Governing flow Equations

The general equations for flow through saturated-unsaturated media are based on: (1) fluid continuity, (2) solid continuity, (3) fluid movement (Darcy’s law), (4) stabilization of media, and (5) water compressibility [28]:

(1) $$\frac{\rho }{\rho_{o}}F\frac{\partial h}{\partial t}=-𝛻\cdot \left(\frac{\rho }{\rho_{o}}V\right)+\frac{\rho^{*}}{\rho_{o}}q,$$

where *F* = a generalized storage coefficient (1/*L*), defined as:

(2) $$F=\alpha^{\prime }\frac{\theta }{n_{e}}+\beta^{\prime }\theta +n_{e}\frac{dS}{dh},$$

*K* = the hydraulic conductivity tensor (*L*/*T*), defined as:

(3) $$K=\frac{\rho g}{\mu }\;K=\frac{{\rho /\rho }_{O}}{\mu /\mu_{O}}\frac{\rho_{o}g}{\mu_{O}}\;K_{s}K_{r}=\frac{\left(\rho /\rho_{o}\right)}{\left(\mu /\mu_{o}\right)}K_{\mathrm{so}}k_{r},$$

and *V* = Darcy’s velocity (*L*/*T*), described as:

(4) $$V\;=-K\cdot \left(\frac{\rho_{o}}{\rho }𝛻h+𝛻z\right),$$

where ρ is the fluid density [M/L^3^], $\rho_{o}$ is the reference fluid density at zero chemical concentration and at the reference temperature [M/L^3^], *F* is the generalized storage coefficient [1/L], *h* is the pressure head [L], *t* is time [T], $\rho^{*}$ is the fluid density of either injection $\left(\rho^{*}=\rho \right)$ or withdrawal (=ρ) [M/L^3^], *q* is a source or sink representing artificial injection or withdrawal of fluid [(L^3^/L^3^)/T], *V* is the specific discharge or Darcy’s velocity [L/T], *K* is the hydraulic conductivity tensor [L/T], *z* is the potential head [L], $\alpha^{\prime }$ is the modified compressibility of the media [1/L], θ is the effective moisture content [L^3^/L^3^], n_e_ is the effective porosity [L^3^/L^3^], $\beta^{\prime }$ is the modified compressibility of the liquid [1/L], *S* is the degree of saturation of water, *g* is acceleration due to gravity (L/T^2^), $\mu_{o}$ is the fluid dynamic viscosity at zero chemical concentration and at the reference temperature M/(L/T), μ is the fluid dynamic viscosity M/(L/T), *K_so_* is the reference saturated hydraulic conductivity tensor [L/T], and *k_r_* is the relative permeability or relative hydraulic conductivity (dimensionless). 

The finite element method is used to solve Equations (1)–(4), and the constitutive relationships between pressure heads, hydraulic conductivity tensor, and degree of saturation along with the appropriate initial and boundary conditions. The initial conditions for this study were obtained from field measurements. In the case of transient simulation, the initial conditions must be realistic and consistent. Initial conditions that are not appropriate are likely to introduce either non-convergence or non-realistic solutions, and therefore these conditions need to be specified as close to the actual situation. In a number of situations, it may be impossible to measure the initial pressure field across an entire study domain. In such situations, an alternative way of setting the initial conditions is to assume that, in general, steady-state flow conditions may have existed. Therefore, the simulation results from a steady-state simulation with steady-state-specified boundary conditions used.

#### 2.2.3. Governing Reactive Transport Equations

The governing equations for the reactive transport of the reactive biogeochemical system are discussed below. The governing equations for transport were derived based on the continuity of mass and Fick’s flux laws [22,28]. The main transport processes are advection, dispersion and diffusion, source and sink and biogeochemical reactions (including radioactive decay). The general transport equation governing the temporal-spatial distribution of any biogeochemical species in a reactive system is described below. Let *C_i_* be the concentration of the *i*^th^ species; then, the governing equation for *C_i_* is obtained by applying the principle of mass balance in integral form, as follows [28]:

(5) $$\frac{\partial \theta C_{i}}{\partial t}+\theta \alpha^{\prime }\frac{\partial h}{\partial t}C_{i}=L\left(C_{i}\right)+\theta r_{i}+M_{i},i\in \left\{M\right\}$$

where *L* is the transport operator denoting:

(6) $$L\left(C_{i}\right)=-𝛻•\left(VC_{i}\right)+𝛻•\left[\theta D•𝛻C_{\begin{array}{c} i \end{array}}\right]$$

(7) $$\frac{D}{Dt}\int_{v}\theta \;C_{i}dv=-\int_{\Gamma }n•\left(\theta \;C_{i}\right)\;V_{i}d\;\Gamma -\int_{\Gamma }J_{i}d\;\Gamma +\int_{v}\theta \;r_{i}dv+\int_{v}M_{i}dv,\;i\;\in M\;$$

where *C_i_* is the concentration of the *i*^th^ species in moles per unit volume [M/L^3^]; *ν* is the material volume containing a constant amount of media (L^3^); Γ is the surface enclosing the material volume *ν* (L^2^); *n* is the outward unit vector normal to the surface Γ; *r_i_* is the production rate of the *i*^th^ species due to biogeochemical reactions, in chemical mass per water volume per unit time [M/L^3^/T]; {M} = {1,2,…,M}, in which *M* is the number of biogeochemical species; *D* is the dispersion coefficient tensor [L^2^/T]; and *M_i_* is the source/sink of the *i*^th^ species in chemical mass per unit volume of media [M/L^3^/T]; *M* is the number of biogeochemical species; *v_i_* is the transporting velocity relative to the solid of the *i*^th^ biogeochemical species (L/T); *θr_i_* is the production rate of the *i*^th^ species per unit medium volume due to all biogeochemical reactions [(M/L^3^)/T], *Ji* is the surface flux of the *i*^th^ species due to dispersion and diffusion with respect to the relative fluid velocity [(M/T)/L^2^] and *Vi* is the transporting velocity relative to the solid of the *i*^th^ biogeochemical species (L/T). 

As in the flow model, in order to simulate reactive transport across a wide range of problems, appropriate transport boundary conditions were applied in the model. The physical definitions and mathematical descriptions of these boundary conditions are comparable to those of the flow model.

#### 2.2.4. Equations for Geochemical Processes

The production of a species along its transport path is governed by a number of biogeochemical processes. One of the difficult aspects of geochemical modelling is the formulation of a governing rate equation to represent the chemical processes governing the rate of production of any species (*r*_i_ in Equation (5)) and its associated parameters. The formulation of rate equations related to all *N* reactions is a critical issue in the modelling of mixed equilibrium and geochemical kinetic reactions. A rate equation is essential for the quantitative description of a general geochemical reaction that is written as follows [28]:

(8) $$\underset{i\in \left\{M\right\}}{\sum }\mu_{ik}{\hat{C}}_{i}↔\underset{i\in \left\{M\right\}}{\sum }\nu_{ik}{\hat{C}}_{i},k\in \left\{N\right\}$$

where ${\hat{C}}_{i}$ is the chemical formula of the *i*^th^ species; *μ_ik_* is the reaction stoichiometry of the *i*^th^ species in the *k*th reaction associated with the reactants; *ν_ik_* is the reaction stoichiometry of the *i*^th^ species in the *k*th reaction associated with the products; and {N} = {1, 2, …, N}, in which *N* is the number of reactions.

For all geochemical reactions, two categories of reactions exist: kinetic and equilibrium. Assuming that there are NE fast/equilibrium reactions (all of which must be independent) and NK slow/kinetic reactions [16], then the number of reactions will be: N = NE + NK.

Due to the nature of the study area and its complexities, a reaction-based formulation was chosen to represent all the geochemical processes in the aquifer site. In a reaction-based formulation, all biogeochemical processes are conceptualized and transformed into a reaction network [28]. This is to consider the contributions of the individual process reaction interplays in the aquifer system and avoid the possibility of the production rate being represented as a lumped rate of all reactions of a particular process which, in this specific instance, will not identify individual reaction rates.

The difficulty in applying the reactive transport model to real-world problems is in transforming the understanding of biogeochemical processes into reaction networks with a rate equation for each reaction. The transformation is a complex task without which our understanding of the aquifer system will be incomplete or inadequate.

### 2.3. Solution Technique

Equations governing the flow and reactive transport processes are a set of partial differential equations that require solving and coupling through flow and transport solutions. The linearized matrix equations can be solved by using the finite element method by applying a number of numerical schemes. A two-step method was used to solve the chemical transport equations and chemical equilibrium equations. Once the solutions for a specific time-step converges, the calculation continues to the next time step [22]. 

Finite element methods were used for temporal discretization of the governing partial differential equations in the flow model and reactive transport model. The Galerkin finite element method was used for spatial discretization of the modified Richards equation. Meanwhile, scalar reactive transport equations were solved using both conventional finite element methods and hybrid Lagrangian–Eulerian finite element methods for spatial discretization. The solutions to the chemical equilibrium equations were obtained using the Newton–Raphson or Picard methods. Three numerical schemes (iteration approach, operator splitting approach, and predictor–corrector approach) were used to couple the hydrological transport and geochemical reactions. Since this study involves a complex three-dimensional model containing more than 6000 elements, 4000 nodes and 33 species, it was more efficient to solve it using the operator splitting approach. Hence, coupling of the transport and geochemical reactions was achieved using the operator splitting approach.

The first phase of the adopted methodology consists of a numerical simulation of the physical processes of flow and reactive transport in the groundwater system. To solve the source identification problem, the governing equations of groundwater flow and transport are solved to accurately represent the flow and transport processes occurring in the contaminated aquifer system. The simulation of these processes requires data on the hydraulic head fields and contaminant concentrations so that the governing groundwater flow and transport equations, respectively, can be solved. Multiple reactive transport and mixtures of contaminant plumes in an aquifer present an intricate problem likely influenced by, but not limited to, complexation, precipitation–dissolution, adsorption–desorption, advection, dispersion and diffusion, and the chemical processes of aqueous, ion-exchange, redox and acid–base reactions.

This study used contaminant concentration measurements for the mine site that were obtained by a previous study [29,30,31]. These data are very limited and only cover two years of monitoring. The concentration calibration results of the present study were compared with these limited measurement data. No other concentration data were available. This study illustrates the limitations in modelling flow and transport processes at such a site with very limited field measurements. The limitations in concentration data, and the very limited knowledge on aquifer parameter values, were a challenge to the development of a well-calibrated simulation model. Such a challenge is common in this area of research. This is also one of the reasons why a large number of modelling iterations with different assumptions of recharge, boundary conditions, and initial head and concentration values was needed to achieve an acceptably calibrated model. The implementation of flow and transport simulation models should be considered in light of such limitations and the challenges common to contaminated aquifer sites such as the current one.

### 2.4. Conceptual and Numerical Development

#### Conceptual Approach Overview

A numerical groundwater flow model was constructed to simulate the groundwater flow system at the Rum Jungle Mine site from 2010 to 2012 [32]. This numerical flow model is a mathematical representation of a conceptual model that enables a quantitative representation of real field features. The numerical representation is based on the assumption that the aquifer system at the mine site is subdivided into hydro-stratigraphic units that represent the waste rock dumps and naturally occurring bedrock aquifers. Each hydro-stratigraphic unit is characterized as a single model layer with representative hydraulic properties. Recharge is estimated as a percentage of incident rainfall assigned to infiltration sections at the site based on elevation. The waste rock dumps and open pits represent a single top layer of variable thickness. The other geological aquifer units are represented as model layers with constant thickness across the model domain. The flooded open pit is represented by specified head boundary conditions that are equivalent to the water levels observed in the pits during the simulation period. Groundwater movement in the hydro-stratigraphic units follows Darcy’s law. 

### 2.5. Numerical Model Setup

#### 2.5.1. Flow Model

The finite element method was used for temporal discretization of the underlying partial differential equations in the flow model. The finite element mesh generated for the numerical model consists of 6587 nodes and 10,704 elements. The Galerkin finite element method was used for spatial discretization of the modified Richards equation governing the pressure fields. The numerical model starts at year 2010 because this is when data became available and ends in 2012. Therefore, the numerical model covers a period of approximately 730 days. For time discretization, time steps of 30 days were set. This time discretization criterion resulted in a total of 24 time steps.

**Figure 1:**
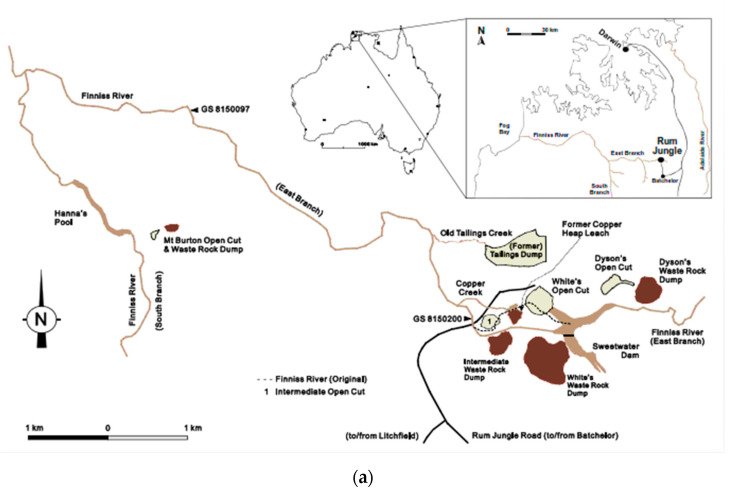

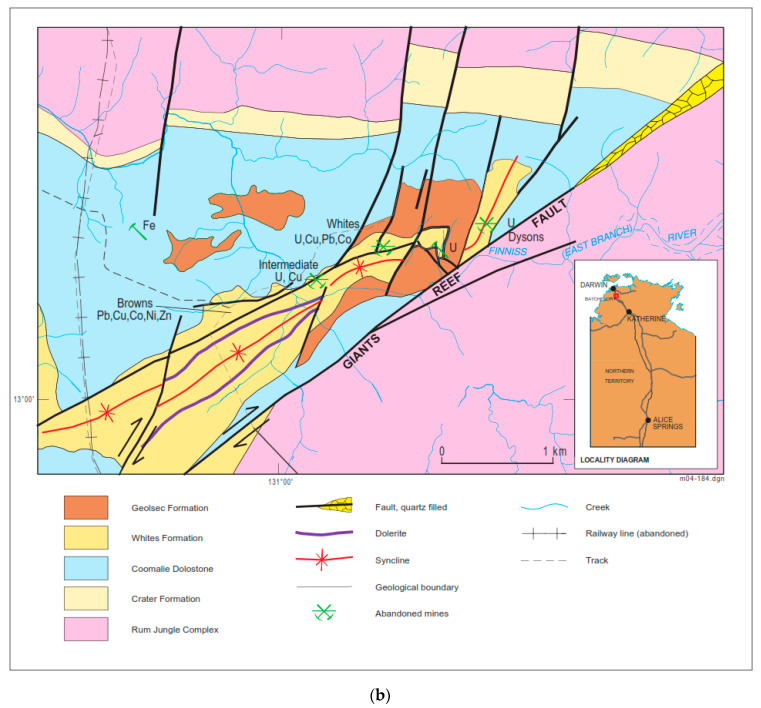
(**a**) A layout map of the Rum Jungle Mine site [33]. (**b**) A geological and mineral deposits map of the Rum Jungle Mine area [34].

**Figure 2:**
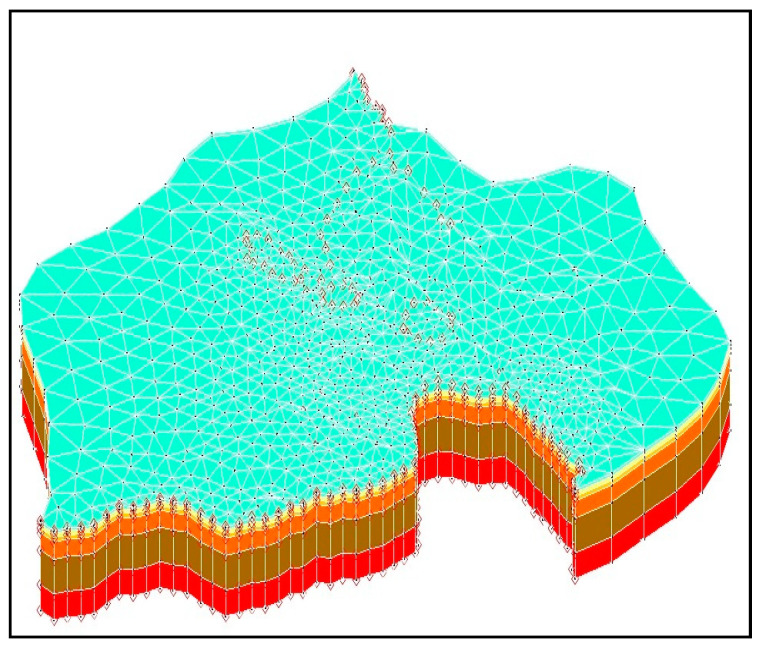
Three-dimensional representation of the model’s boundary conditions.

The numerical model domain was spatially discretized into a three-dimensional mesh with a triangular wedge mesh. In planar view, each element is represented triangularly, whereas the thickness of the elements depends on the number of layers used to vertically discretize the model domain. For this model, the elements in layers were assigned a set of hydraulic properties based on different material types to represent the complex heterogeneity of the aquifer. The thickness of the elements varies according to lithology. The model is made up of six layers and covers a maximum elevation of approximately 110 m. Surface topography elevation values obtained from a digital elevation model (DEM) were used to define the top of Layer 1. Figure 1a shows an aerial layout of the Rum Jungle Mine with the various mine sections and Figure 1b shows details of the geology and mineral deposits. Figure 2 shows the discretization and resulting finite-element mesh in spatial discretization process performed for the numerical model. Layer thicknesses are vertical in depth, with an overall model thickness of 150 m. Layer thicknesses were assigned as follows. Layer 1, which mainly consists of waste rock dumps and tailings, was assigned variable thickness. Layer 2 thickness = 0–7.5 m, Layer 3 = 7.5–15 m, Layer 4 = 15–45 m, Layer 5 = 45–105 m, and Layer 6 = 105–150 m. The tops and bottoms of Layers 3 to 6 were set to the thickness values listed above and were fixed throughout the modelling process.

#### 2.5.2. Boundary Conditions

Several boundary conditions were set for modelling the flow and transport processes of this study area. Specified heads were set to element nodes that interconnect the perimeters of the flooded pits in Layers 2 and 3. Elements and nodes surrounding the pits representing the bedrock aquifer in contact with water inside the pits were set to head values equal to the measured groundwater level in the pits. The model does not simulate flows within the flooded open pits themselves, so elements within the head boundary are not active. Pit water levels and groundwater levels at monitoring points nearby are adapted to represent the open pit as a head boundary condition derived from the groundwater levels measured in monitoring points situated nearer to this boundary. The northern boundary where the Finnish River is located was assigned a constant head boundary condition, as were the creeks alongside the Finnish River’s east side and the creeks at the southern boundaries of the model. For these boundaries, river-bed elevations, and temporally varying river head values, obtained through field observations, were assigned. A transient constant head boundary was set that simulates groundwater water-level changes in the main, intermediate and brown pits, and the river. The finite element method provided ease of using variable meshes, ease of incorporating all the boundary conditions and accurate geometric representations of the aquifer system. The discretization of the study area and model boundary conditions is shown in Figure 2.

#### 2.5.3. Model Input Parameters

Several input parameters for aquifer properties and boundary conditions were used to broadly describe characteristics of the aquifer. Aquifer properties defines the geological medium through which groundwater flows in terms of porosity, hydraulic conductivity, bulk density, and moisture content, while groundwater boundary conditions describe the water flux between aquifer layers and surface features such as groundwater recharge rate and well pumping schedule. The hydraulic conductivity values and specific yield/specific storage values used for the model’s hydro-stratigraphic units were estimated from pumping tests described in previous studies [34]. 

The initial hydraulic head for the model was based on groundwater-level data measured at 22 monitoring wells in August 2010. The initial hydraulic head ranged from 50 to 71 m Australia height datum (mAHD). Groundwater recharge was estimated from annual rainfall data recorded at the site. It was assumed that net recharge by rainfall and flows from the flooded open pits were the only sources of water input to the groundwater system within the model domain. The aquifer is recharged largely by rainfall and sources of surface water bodies. The rainfall distribution acrosss the study area was estimated from annual rainfall records for the study area, which was 2372 mm per year. Based on calibration and validation of the flow models, the vertical annual recharge was assumed to be 25% of the average gross rainfall over the study area. Groundwater flow processes were simulated using the hydrogeological parameters listed in Table 1. The vertical and horizontal hydraulic conductivities (Kx and Kz, respectively) are shown in Table 2 for the different geological layers of the study area. The hydraulic conductivity in the other horizontal direction, Ky, was assumed to be same as the horizontal hydraulic conductivity Kx.

#### 2.5.4. Reactive Transport Model

To represent the reactive geochemical processes that occur at the study site, a set of reaction networks consisting of equilibrium and kinetic reactions that describe the aquifer’s geochemical processes was formulated, based on contamination and groundwater quality data [10]. HYDROGEOCHEM 5.0 was used to simulate reactive chemical transport and the long-term behaviour of contaminant movement in the aquifer. This study considered the hydrogeochemical transport of six components—OH^−^, Cu^2+^, Fe^2+^, Fe^3+^, Mn^2+^ and UO_2_^2+^—and an aqueous complexation of 17 species and three minerals (pyrite, uranite and chalcopyrite). Geochemical reactions of precipitation and dissolution aqueous complexation, and mineral dissolution were also incorporated into the transport model. 

Since the actual fluxes of the six assumed sources of contamination (four waste rock dumps and two pits) are not known, the groundwater contaminant concentrations measured at several monitoring locations were used as initial concentration data. The first step of contaminant transport modelling is to observe how the contaminant spreads over the field with varying groundwater heads. The groundwater contaminant concentrations measured at some monitoring locations were used in the reactive transport modelling. These concentrations were constant at selected monitoring points on specific days. Additionally, some of these contaminant concentrations were quite low and tended to decrease very quickly or even almost disappear after a few days, probably due to nominal immediate dilution, which is not realistic. To properly model and achieve realistic modelling, it was necessary to interpolate the known concentration data for the whole study area before using them in transport modelling. Hence, an interpolation of the concentrations was performed for the model domain area before conducting the contaminant transport modelling. Only a few points, which were unevenly scattered across the site, were available for the interpolation, but this is quite typical in hydrogeological studies.

The reason for interpolating contaminant concentrations is to provide a more realistic contaminant distribution as compared to a concentration values scattered even in a non-contaminated area whereas addressing the problem of concentration dilution. Furthermore, there was only one concentration measurement available for each location and each contaminant. Even if this value was entered as a transient contaminant concentration, it would be interpreted as a constant concentration and cause the modelling of the contaminant process to be insignificant. Thus, even in a large time-scale simulation, such as that used in this study, contaminant transport would not be detectable. The contaminant concentration data obtained from interpolation were used only in the contaminant transport model as starting concentrations. Thus, the interpolated concentration data were used as initial concentrations. The reactive transport model was simulated (run) for a period of two years (from 2012 to 2014) due to the availability of data. Assumed initial conditions for contaminant concentrations were specified in the simulation model based on groundwater quality data from 2011. Copper (Cu^2+^), sulfate (SO_4_^2−^), manganese (Mn^2+^), uranium (UO_2_^2+^) and iron (Fe^2+^) were introduced as initial contaminants in this study. 

#### 2.5.5. Conceptual Reactive Transport Modelling 

The reactive transport model for the study area was built upon the flow model by implementing the necessary transport conditions. These include the transport boundary conditions, the total number of components and the species to be simulated. The assumption is that the transport of contaminants is based solely on the simulated flow fields, which may involve waste rock interactions at the four waste rock dumps (Figure 1) or mineral interactions with the aquifer rock bed and formation of daughter products or additional metal species. 

The conceptual model for the reactive geochemical system is based on equilibrium and kinetic reactions. The reactive system is entirely described by identifying chemical reactions and the total number of chemical species involved in them. The standard equilibrium reactions with appropriate equilibrium constant is used to represent all the fast reactions, such as aqueous complexation reactions and the precipitation of secondary phases. Slow reactions are represented by kinetic reactions and associated rate constants. This addresses the dissolution reactions involving the major minerals occurring in the waste rock dumps and pits. The reaction network describing the geochemical system of the study area, and the associated rate constants obtained by modifying Yeh [16] equations, are shown in Table 3.

### 2.6. Model Calibration

The groundwater model was calibrated by iterative adjustment of aquifer parameters and stresses to achieve the best match between the observed and simulated water levels. A well-calibrated model accurately replicates hydrogeological conditions of real-world, which is the first goal of modelling. Therefore, the calibrated flow model can provide confidence in the predicted impacts on changes to the groundwater regime. 

### 2.7. Validation of Flow Model

To verify the performance of the groundwater flow and transport models, the transient groundwater model was run for an extended two-year simulation period to replicate groundwater levels from 2012 to 2014/2015. The developed flow model’s outputs (hydraulic heads) were verified against field data from 2014/2015. The model was validated by comparing the solutions of the calibration simulation with a separate set of field measurements not utilized for calibration. It was not possible to calibrate the contaminant transport model as the contaminant source magnitudes, timings and locations could not be specified accurately. The results obtained for this validation exercise are presented; they match satisfactorily with field measurements and thereby demonstrate good predictive ability. However, based on approximate assessment of the sources, the simulated concentrations were informally compared with measured concentrations to ascertain, if the simulated concentrations patterns are similar to the observed concentrations. 

### 2.8. Adaptive Simulated Annealing Optimization Algorithm (ASA)

The second step involves using an optimization algorithm to find optimal candidate solutions. The present study uses an ASA optimization algorithm in the optimal source characterization model. This algorithm is preferred for its comparative efficiency in reaching a global optimal solution. The optimization algorithm is used hereafter to minimize the objective function formulation.

The ASA global optimization algorithm relies on random importance-sampling of parameter space [24]. It was created with the objective of speeding up the convergence of standard SA methods [25]. The basic structure of the ASA algorithm is the same as that of classical SA. There are, nevertheless, some key differences. It has new distributions for the acceptance and state functions and a new annealing schedule. It uses independent temperature scales for each fitted parameter and for the acceptance function. It also performs reannealing at specific intervals. The ASA algorithm maintains the advantages of SA but converges faster. Hence, the ASA algorithm is a powerful global optimization tool for solving complex parameter estimation problems.

The major advantage of ASA is that the algorithm parameters are modified in an adaptive way and that the solutions do not differ greatly if the parameters are updated within acceptable limits. This contrasts with other optimization algorithms, where only small variations in parameters, such as mutation probability, crossover probability, or population size, cause major differences in solutions [25]. An additional benefit of ASA over SA is that it overcomes the speed issue of traditional SA approaches and ensures fast convergence towards a global minimum solution. 

### 2.9. Source Identification Using Simulation Optimization

The simulation-optimization model simulates the physical processes of flow and reactive transport within an optimization model. The flow and reactive transport simulation models are considered as important binding constraints for the optimization model. In this identification model, the flow and transport simulation models are linked to the optimization model using the ASA algorithm to find the solution. 

In simulation-optimization models, the groundwater contaminant source identification problem is formulated as a forward-time simulation in combination with an optimization model. The simulation-optimization model simulates the physical processes of flow and reactive transport within the optimization model. The flow and reactive transport simulation models are treated as important binding constraints for the optimization model to ensure that the simulated source responses fare properly simulated. Therefore, any feasible solution of the optimization model is based on the implemented flow and transport simulation models. The advantage of this approach is that it becomes possible to incorporate any complex numerical simulation model to the optimization model. 

**Figure 3:**
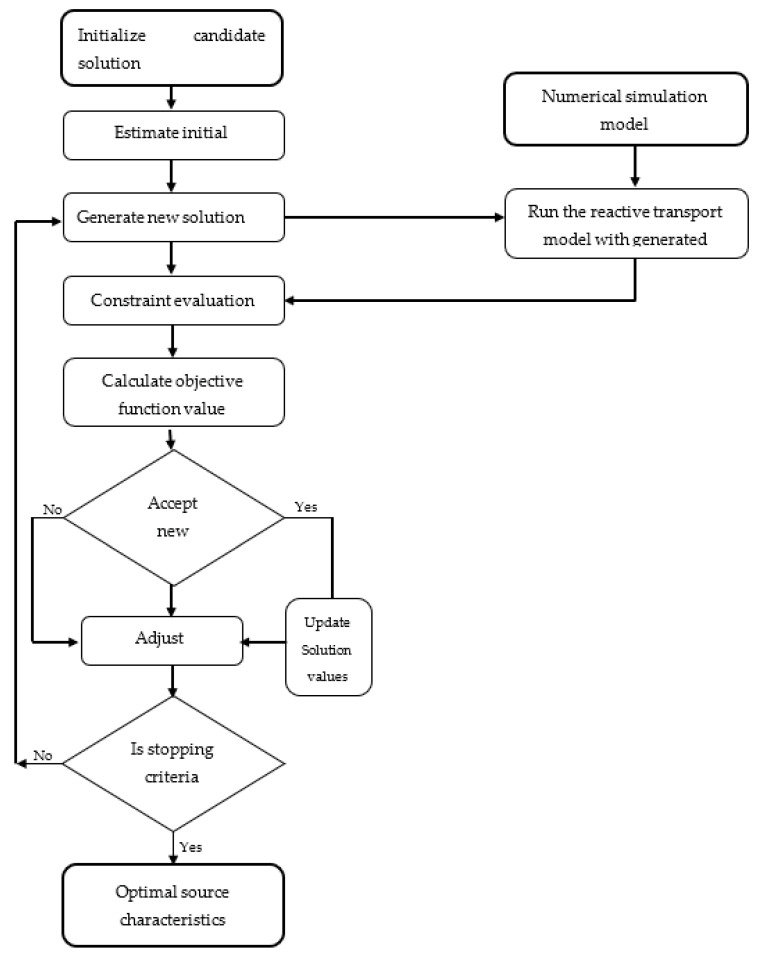
Schematic illustration of the linked simulation-optimization model using adaptive simulated annealing.

In the source identification model, the flow and transport simulation models are linked to the optimization model using the ASA algorithm to find a solution. Figure 3 shows a flowchart of the simulation optimization and program execution sequence for groundwater contaminant source identification.

### 2.10. Mathematical Formulation of Simulation-Optimization Models

The main goal of the contaminant source identification model is to characterize each source according to the geochemically reactive species that are reacting in the aquifer. The source characteristics of interest include location and release duration and magnitude [35,36]. The source identification model uses the optimization approach to provide candidate solutions for a set of source characteristics. It minimizes a weighted objective function of the differences between the simulated and observed species concentrations at monitoring locations within the model domain. 

The optimization model generates candidate concentrations of species associated with each potential distributed source location. In this case, five candidate solutions of species concentrations are generated by the optimization algorithm at six separate potential distributed source locations. These candidate concentration solutions generated by the optimization algorithm are utilized to estimate spatial and temporal contaminant species concentrations in different time periods for monitoring locations at which field concentrations have been measured. Appropriate constraint conditions can be imposed on the parameters of the model. The optimization algorithm then evaluates the objective function. The objective function value is defined as a function of the differences between the observed and simulated concentrations of different reactive species at monitoring locations in different time periods. Optimal source characterization is obtained by solving the optimization model to minimize the objective function. The objective function of the simulation-optimization model used for source characterization was formulated as follows [10]:

(9) $$\begin{array}{cc} Minimize\;F & =\overset{n_{k}}{\underset{k=1}{\sum }}\overset{n_{ob}}{\underset{iob=1}{\sum }}{\left(C_{sp1}{}_{obs_{iob}}^{k}-C_{sp1}{}_{sim_{iob}}^{k}\right)}^{2}•w_{iob}^{k}+\overset{n_{k}}{\underset{k=1}{\sum }}\overset{n_{ob}}{\underset{iob=1}{\sum }}{\left(C_{sp2}{}_{obs_{iob}}^{k}-C_{sp2}{}_{sim_{iob}}^{k}\right)}^{2}•w_{iob}^{k}\\ & +\overset{n_{k}}{\underset{k=1}{\sum }}\overset{n_{ob}}{\underset{iob=1}{\sum }}{\left(C_{sp3}{}_{obs_{iob}}^{k}-C_{sp3}{}_{sim_{iob}}^{k}\right)}^{2}•w_{iob}^{k}+\overset{n_{k}}{\underset{k=1}{\sum }}\overset{n_{ob}}{\underset{iob=1}{\sum }}{\left(C_{sp4}{}_{obs_{iob}}^{k}-C_{sp4}{}_{sim_{iob}}^{k}\right)}^{2}•w_{iob}^{k} \end{array}$$

(10) $$\mathrm{Subject}\;\mathrm{to}:\;C_{sim_{iob}}^{k}=f\left(x,y,z,C_{sim}\right)$$

(11) $$\mathrm{The}\;\mathrm{weight}\;w_{iob}^{k}\;\mathrm{can}\;\mathrm{be}\;\mathrm{described}\;\mathrm{as}:\;w_{iob}^{k}=\frac{1}{{\left(C_{obs_{iob}}^{k}+\eta \right)}^{2}}$$

where:

$C_{sp}{}_{obs_{iob}}^{k}$ is the observed concentration of a species at monitoring location *iob* in the *k*th time period; $C_{sp}{}_{sim_{iob}}^{k}$ is the concentration of a species estimated by the source identification model at monitoring location *iob* in the *k*th time period; *sp1, sp2, sp3, sp4* are species numbers one, two, three and four, respectively, involved in the chemical reaction; n*_ob_* is the total number of concentration observation locations; *nk* is the total number of concentration observation time periods; *nspe* is the total number of species involved; *f (x, y, z, C_sim_)* represents the concentration simulation results at location coordinates defined by x, y, z obtained from the transport simulation model; $w_{iob}^{k}$ is an assigned weight corresponding to observation location iob and time period k; and η is an appropriate constant that is the average of the highest and lowest concentrations of each species. This ensures that errors at low concentrations do not dominate the solution.

Development of a Linked Simulation-Optimization Model for Source Characterization based on Multiple Species of Contaminants

A finite element-based three-dimensional numerical simulator (HYDROGEOCHEM 5.0) was used to simulate the flow and transport processes in the study area aquifer. Hydrological variables, including Darcy’s velocity and moisture content, are necessary in determining the transport of contaminants through saturated-unsaturated subsurface systems. These variables need to be specified to solve the basic governing equations in a simulation model. These variables can be iteratively estimated by calibration of a simulation model. 

A linked simulation-optimization approach linking the groundwater numerical simulation model with an optimization model that incorporates an ASA algorithm was implemented. Integrating a numerical simulation model with an optimization algorithm results in significant performance improvement over source identification results obtained through conventional standalone simulation or optimization methods. In the linked simulation-optimization methodology, the first step is to develop a groundwater simulation model that computes head and species concentration values at different monitoring locations in different time steps. In the second step, the simulation model is externally linked with the optimization model. Whenever the optimization procedure requires the objective function and/or constraint evaluation, it calls the simulation model while passing the candidate solutions to the simulator. Then, the simulation model executes and returns the resulting concentrations. An ASA algorithm acts as a driver model that calls the simulation model by passing variables and gets back the corresponding objective function value. The ASA then adjusts the variables to compute a new objective function and continues for several iterations until there is no further improvement or the stopping criteria are satisfied. The ASA used as the optimization routine calls the calibrated simulation model during each iteration.

A computer code was written in C++ language to interface the ASA and calibrated groundwater simulation model, thus facilitating communication between the simulation and optimization models. The C++ based code acts as a subroutine that has a set of instructions designed to communicate between the FORTRAN-based numerical simulation program and the C-language-based optimization program of ASA to perform frequently used operations within the linked simulation-optimization methodology. 

For the simulation and optimization models to work efficiently together to optimally solve the source identification problem, they must be interfaced. This requires the design and implementation of a linking script that facilitates communication (recursive calls) between each module, hence the C++ code. Each time an optimization model requires a function evaluation or constraint evaluation, it calls the simulation model. Figure 1 shows the program’s execution sequence. The ASA algorithm starts from an initial guessed solution given by the user. Candidate source concentrations are generated randomly by the ASA algorithm as possible solutions. For each species’ set of source concentration values, the numerical simulation is executed once to update the concentration in response to the source concentrations. The output of the simulation consists of concentration values for all nodal points in the simulation domain. The concentration values for the selected observation locations are forwarded to the optimization module. The optimization algorithm evaluates the constraints and checks for termination. If the constraints are not satisfied, then it computes new source concentrations based on the ASA model and passes them to the numerical simulation model. Based on the HYDROGEOCHEM results, a new set of source concentrations is formed and HYDROGEOCHEM is called again to compute the concentrations. This process is continued until an optimal solution is reached based on the objective function and the constraints. The time period for optimization is given by the user. The numerical model will start running from the initial time period, irrespective of the optimization time period and, through this, the numerical model will take care of the time relationship. HYDROGEOCHEM 5.0 uses many input files, but during optimization, only the candidate source concentrations of individual contaminants change. All other parameters do not change; hence, only the source concentration files are modified using the linking C++ code during each iteration. The idea here is to introduce new candidate solutions through the source concentration files and then run the whole optimization model. The file containing the concentrations of individual contaminant species at different time steps and different observation locations is checked by solving the objective function, whether the constraints are satisfied or not.

### 2.11. Performance Evaluation

To evaluate the performance of the proposed optimal contaminant source characterization methodology, concentration measurements taken at monitoring locations in different time steps were utilized as a part of the real-life illustrative scenario. To test the reliability and robustness of the proposed methodology in real scenarios, synthetic concentration measurement errors were incorporated by introducing various amounts of synthetically generated, normally distributed error in the simulated concentration values [37]. The perturbed simulated concentrations represent erroneous measurements, and are defined as follows:

(12) $$ertC_{sim_{iob}}^{k}=C_{sim_{iob}}^{k}+\varepsilon •a•C_{sim_{iob}}^{k}$$

where:

$pertC_{sim_{iob}}^{k}$ is a perturbed simulated concentration; $C_{sim_{iob}}^{k}$ is a simulated concentration; ε is a normally distributed error term with a zero mean and standard deviation of one; a is a fraction, such that 0 < a < 1. For example; a is varied from 0.05 to 0.2. a < 0.10 corresponds to a low noise level, 0.10 < a < 0.15 corresponds to a moderate noise level, and a. 0.15 corresponds to high noise level [37].

#### Model for Erroneous Concentration Measurement Values

For evaluation purposes, simulated concentration values were perturbed to represent measurement errors. Adding randomly-generated random errors to the simulated concentrations perturbed these simulated values. The normally distributed random error terms are used to simulate the errors that generally occur in field measurements. The perturbed concentration values were computed as follows [38]:

Cobs (*M_L_, t*) = Csim (*M_L_*, t) + *ε r* (13)

where:

*Cobs* (*M_L_, t*) is the measured or observed concentration of a species at monitoring location *M_L_* at time *t*; *Csim* (*M_L_, t*) is the simulated concentration at location *M_L_* and time *t* from the numerical simulation model; and *ε r* = is a random error term. 

Here, the random variable *ε r* is assumed to follow a normal distribution with mean = 0 and standard deviation = *a.Csim (M_L_, t*). Furthermore, the error term is defined as:

*εr* = *e. a. Csim* (*M_L_, t*) (14)

where *a* = a fraction (0 ≤ *a* ≤ 1.0) and *e* = normal deviates.

For this study, MATLAB (R2017a) software was used to generate standard normal deviates (e). The value of a was varied from 0.05 to 0.2, where higher values indicate a higher level of noise in the data. It was assumed that values of a < 0.10 correspond to a low noise level, 0.1 ≤ a ≤ 0.15 corresponds to a moderate noise level, and a > 0.15 corresponds to a high noise level [37,39]. Additionally, for performance evaluation purposes a normal distribution of errors is assumed. Any other suitable distribution function may be incorporated. The value of Cobs (*ML, t*) can be negative if e is negative, a is large and Csim (ML, t) is small. Generally, such a situation is less probable if a is small and e is also small. Otherwise, a truncated normal distribution may be used.

### 2.12. Optimal Source Characterization

The numerical simulation model used to solve the three-dimensional flow and reactive biogeochemical transport processes was utilized in the linked simulation-optimization model for optimal source characterization of different distributed sources in the contaminated mine site area. For evaluation purposes only, concentration measurements at specified monitoring locations were simulated (synthetic measurements) by the calibrated numerical flow and transport simulation model. These concentration data were generated for specified source characteristics. Then, they were used with the linked simulation-optimization models to evaluate the potential applicability, accuracy and feasibility of the developed methodology. After evaluating the performance utilizing synthetic simulated measurement data, the developed methodology was applied to characterize sources based on contamination measurements obtained in the study area. 

However, as is generally the case for such large-scale, complex, contaminated aquifer sites, the contaminant sources are unknown. Hence, the source concentration magnitudes or source activity starting times cannot be validated. Hence, synthetic concentration values were utilized with the calibrated flow model to evaluate the performance of the source characterization methodology. Concentrations of contaminants measured in the field [40] in 2011 and 2012 were used in the source characterization process.

## 3. Results

### 3.1. A Numerical Simulation Model

#### 3.1.1. Calibration of the Flow Model

For a model to be able to adequately and accurately simulate field parameters, it needs proper calibration. The aim of calibrating a model is to tune the hydrogeological parameters until the model approximates field measurements such as hydraulic heads and concentrations. The idea is to simulate the physical processes in the aquifer accurately. The flow model was calibrated for hydrogeological parameters and boundary conditions by running the forward simulation repeatedly and manually adjusting the input parameters selected for calibration, including boundary conditions, within their allowable ranges in each run until a satisfactory match between the modelled and field results was achieved. In this study, a trial-and-error procedure was used. It is worthwhile noting that the numerical simulation codes utilized in this study do not follow an automated calibration procedure such as PEST [41]. Manual trial-and-error calibration runs are conceptually straightforward and require intuitive judgment of the results obtained from multiple forward simulation runs. This process is flexible, allowing logical adjustments of parameter values and structures, including changes in mesh designs and the representation of the geological framework. For this study, the purpose of calibration was to obtain hydraulic conductivity and recharge estimates for the modelled aquifer based on limited field measurements. Calibration was attempted without changing the hydrogeological zones defined by the distribution of hydrogeological units present at the site. Calibration was attained through refinement of the model parameters and features, including hydraulic conductivity in the horizontal and vertical dimensions, and recharge and hydraulic conductivity assigned to the sections used to replicate the influence of the fault.

Since the flow model simulation started in December 2010, groundwater head data measured from 20 monitoring locations in December 2010 were used to calibrate it. Throughout the calibration process, the hydraulic conductivity values of the different soil materials and rock types were varied within the acceptable range of field-measured hydraulic conductivity values in Table 2. Simulation runs were repeated until a reasonable match between the observed and estimated hydraulic head values was reached under the transient-state simulation conditions. Different values of hydraulic conductivity were obtained after calibration. The hydraulic conductivity values differed from layer to layer based on the varying material properties of each layer. Each layer had specific hydraulic conductivity values. The hydraulic conductivity values ranged from 0.1 to 5 m/day with the exception of the fault zone that cuts through the east side of the model, which had values as high as 75 m/day. 

Hydraulic head measurements from 22 observation locations were used to calibrate the simulation model. Data from 2010 to 2012 were used to calibrate the flow model, while data from 2012 to 2014 were used to validate it. For the purposes of calibration, a percentage of annual rainfall was defined as a recharge value in the model. The calibration targets for the developed model were set to be within 2 m of the hydraulic head values observed at the observation locations.

Determining the exact boundary conditions of a model domain is a difficult task when measurement data are limited. Without exact or characteristic boundary conditions, a model may not accurately represent a field process. It is therefore necessary to implement realistic boundary conditions in a model that reflects the conditions of the site. One of the most difficult tasks in the calibration process is to properly assign the correct boundary conditions. The boundary conditions must therefore also be determined on the basis of the preliminary results of the calibration. The boundary conditions of the model are manually adjusted to achieve the calibration targets. In this study, simulated hydraulic heads were compared with field-measured hydraulic heads at monitoring points. Groundwater generally flows from the higher elevations of the study area towards the central area, towards the east branch of the Finniss River northwards. 

**Figure 4:**
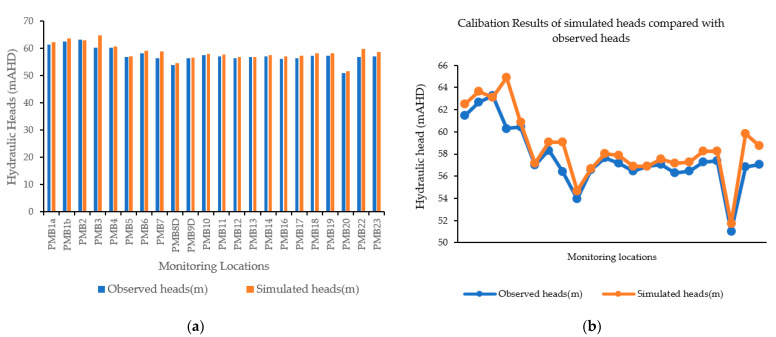
(**a**) Comparison of hydraulic heads observed in 2012 with those estimated by the calibrated model; (**b**) calibrated results of hydraulic heads for 2012.

The hydraulic head calibration results are illustrated by the bar graphs in Figure 4a, which compare the simulated and measured values. The same comparison is presented in Figure 4b but as a line graph. A graph of the simulated and observed groundwater levels at selected monitoring locations after calibration is given in Figure 4. This figure shows the monitoring location of wells on the *x* axis and the head values in metres on the *y* axis. Figure 4b comparse the observed and simulated heads for the two-year period of 2010–2012. Figure 4a,b show that there is an acceptable match between the field-measured and simulated heads. There is a small difference between the measured and simulated heads, which may be due to various errors and uncertainties in measurement, measurement/estimation of parameters, and boundary conditions. Variations within the model elements due to minor deviations in the hydraulic parameters, as is commonly found in groundwater modelling investigations, may be an additional cause. The deviation between the simulated and measured hydraulic heads does not exceed 5% of the field-measured values, so the calibration process can be said to have made the model reasonably approximate the observed groundwater head values. The calibration results, as a comparison between observed and simulated groundwater levels, are shown in Figure 4.

**Figure 5:**
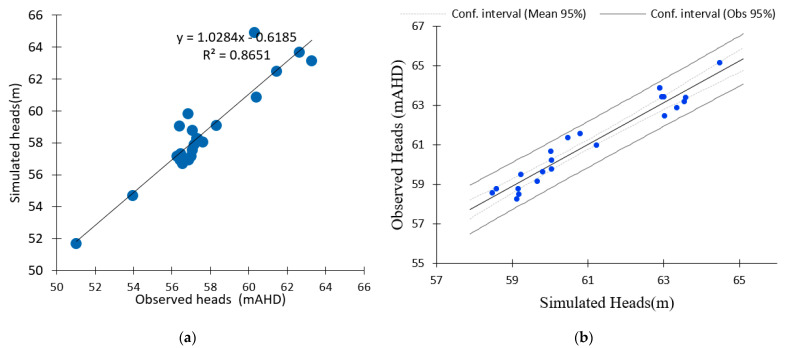
(**a**) Correlation between simulated and observed groundwater heads; (**b**) linear relationship between measured and Scheme 95. confidence intervals for the mean of the observed values and the observed values.

A scatterplot of simulated vs. observed values can be considered as a calibration graph. A plot for the calibration period (2010–2012) is shown in Figure 4 Statistical analysis of the calibrated model results shows that the residual mean (RM) groundwater levels at the monitoring locations during the calibration period ranged from 0.01 to 2.97 m. The mean absolute error (MAE) was calculated as 0.82 m, and the standard deviation is 0.77 m. The normalized root mean squared (NRMS) was 0.09 and the root mean square error is 1.10. The correlation coefficient (*R*) is 0.90. The scatterplot in Figure 5a shows the comparison of observed and simulated hydraulic heads, confirming that the simulated head levels were within an acceptable range of the measured heads. Figure 5b shows the correlation between the measured and simulated heads with 95% confidence intervals on the mean observed and measured values.

The results of calibrating the simulated heads are presented in Table 4. The calibration of a numerical model is typically considered good if the NRMS error is <5%. The computed NRMS values for the simulated heads are well below the target of 5%, suggesting good calibration to head targets and simulated hydraulic parameters. The residual average error for the total head data sampled at the 22 monitoring wells in 2010 is 1.0168. The heads range in value from 50 to 70 m. The simulated heads at the monitoring points were then compared with the observed heads. Figure 4 shows bar charts that indicate a close relationship in the hydraulic heads. A Pearson correlation coefficient test was applied to the calibration results of the total heads. The correlation coefficient (*r*) of 0.918 shows a close linear relationship between the observed and simulated heads.

**Figure 6:**
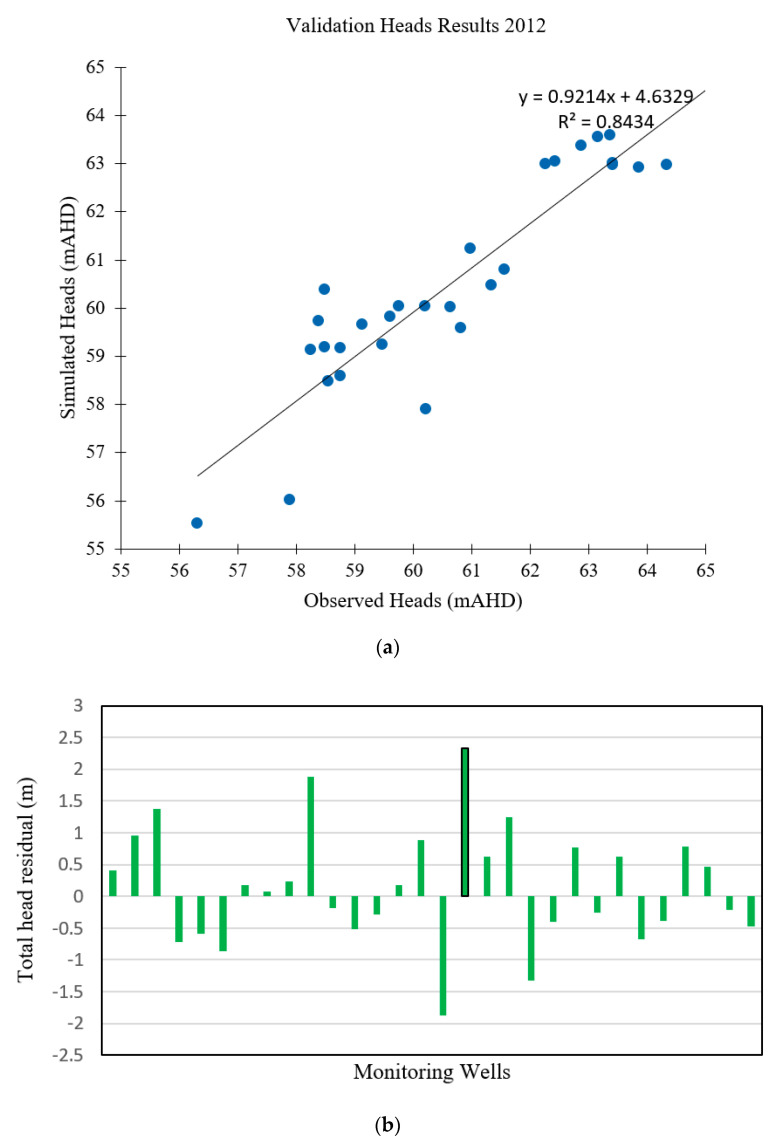
(**a**) Correlation between observed and simulated groundwater levels during the validation period. (**b**) Errors between observed and simulated hydraulic heads during the validation period (2014).

Field data approximately corresponding to the first 738 days were used to calibrate the numerical model at all monitoring points. Field data from after this period were reserved for validating the numerical model. Figure 6 shows the evaluation of the numerical model’s performance in the calibration period. The simulated groundwater heads predict the field-measured data reasonably well for the validation stage. Residuals between simulated and measured groundwater heads were also calculated by means of mean absolute error (MAE), NRMSE and RMSE. Table 5 shows the statistics for the calibration and validation periods. Figure 6a,b show that the numerical model maintains its calibrated accuracy throughout the validation period.

#### 3.1.2. Validation of the Flow Model

The validation results for the validation period of 2012–2014 are shown in Figure 6. Table 4 presents the validation results in terms of hydraulic heads. 

The availability of hydraulic head field measurements from 2012 to 2014 allowed the model to be validated over this period, using the 2012 measured head distribution as the initial condition. Simulation was carried out until 2014 using a time step period of 30 days. Figure 6 compares the hydraulic heads observed and simulated for this period, illustrating a wide correspondence. Thus, after transient-state validation, the model is shown to simulate groundwater levels with a reasonable level of accuracy.

The validation residual results representing the differences between the observed and simulated groundwater levels are given in Figure 6. This graph shows that the residuals were typically less than 1 m (with the average depth of the aquifer being approximately 150 m) throughout the majority of the model domain, except in four monitoring well locations: PMB2, PMB9D, PMB16 and PMB 18. These had larger head residuals and tended to be in areas with a higher topographic relief and/or deeper water table, factors that tend to cause high seasonal fluctuations in groundwater levels. In general, these areas were more difficult to calibrate. Nevertheless, the spatial bias in head residuals was considered acceptable for the purposes of this study.

A summary and comparison of the calibration and validation results are given in Table 5. The maximum deviations in the predicted and measured hydraulic heads are 0.07 m and 0.95 m, respectively. Table 5 also shows that the deviations between the measured and predicted heads are more or less of the same order for both the calibration and validation results. Therefore, given the situation of having limited measured data and a large and hydrogeologically complex study area with a multilayer aquifer, these validation results show that the calibrated model can be utilized for the prediction of hydraulic head under different input scenarios.

The numerical flow model for the Rum Jungle Mine site was calibrated using annual groundwater-level data measured at a selection of bores since August 2010. Moreover, the validation process suggests that the current calibration provides a reasonable approximation of current flow conditions at the Rum Jungle Mine site and can be used to predict the response of the groundwater system for rehabilitation planning.

Concerning the calibration process, it is worth noting that the calibrated values of hydraulic conductivity lie within the range of uncertainty in the values obtained by means of hydrogeological characterization. Average hydraulic conductivity values were used due to the complexity of the aquifer system. The nature of the aquifer requires that a layer has approximately five different conductivity values, which causes convergence issues. The model encountered challenges in converging when 14 distinct hydraulic conductivity values were applied, as average values were used to represent the heterogenous and anisotropic nature of the site as much as possible and to achieve model convergence. 

### 3.2. Optimization Model

The aim of the performance evaluation process was to evaluate whether the source characterization model can recover the actual source characteristics based on synthetic concentration values [42]. Hence, in the linked simulation-optimization model for source characterization, the calibrated numerical simulation models were used to generate synthetic (simulated) concentration data. The reason for using synthetic data for performance evaluation was to ensure that unknown errors in the actual measurements did not distort the performance evaluation results. Additionally, by using synthetic concentration data, it is possible to test the performance with different scenarios of measurement error. Furthermore, the actual sources may not be known, so the performance is evaluated with synthetic concentrations generated for specified contaminant sources. This is to ensure that the estimation results can be verified. Therefore, the source characteristics recovered can be verified from the specified sources used to generate the synthetic data. If the performance is satisfactory, the methodology should be useful for recovering the actual source characteristics.

#### 3.2.1. Case Studies for Model Demonstration

In the following sections, the model for identification of groundwater contaminant sources is demonstrated using two case studies involving a mining area; (i) with simulated (synthetic) measurements for testing (where the sources are known); and (ii) same study area with observed concentrations to demonstrate practical applicability.

##### Calibrated Model Testing with Simulated Data

**Figure 7:**
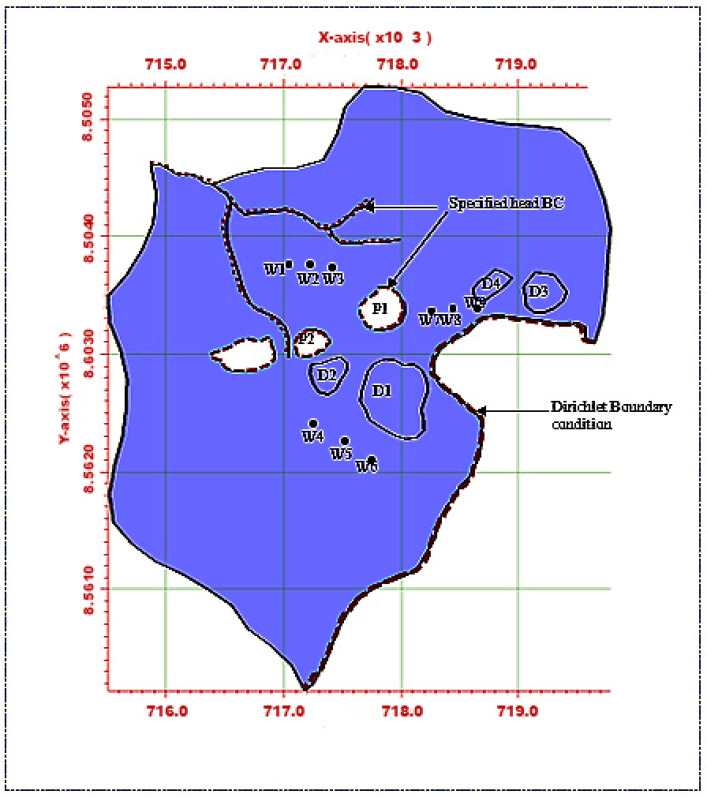
Mine site aquifer with four contaminated waste rock disposal sites and nine observation wells.

**Figure 8:**
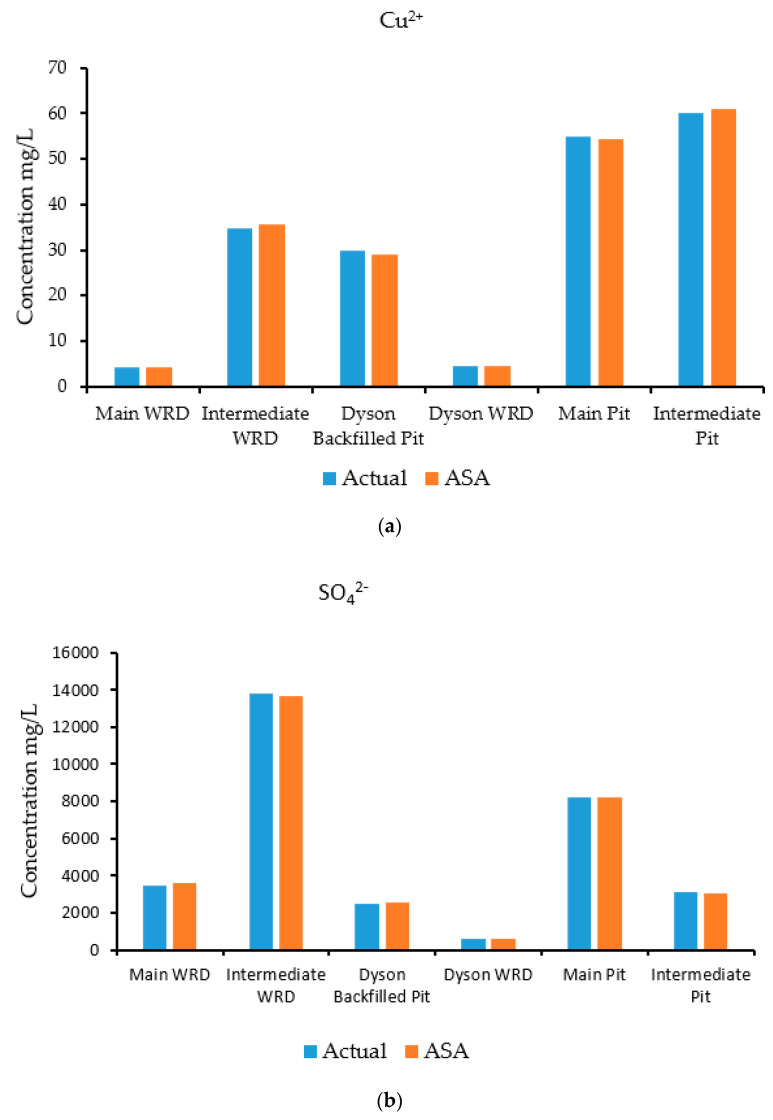

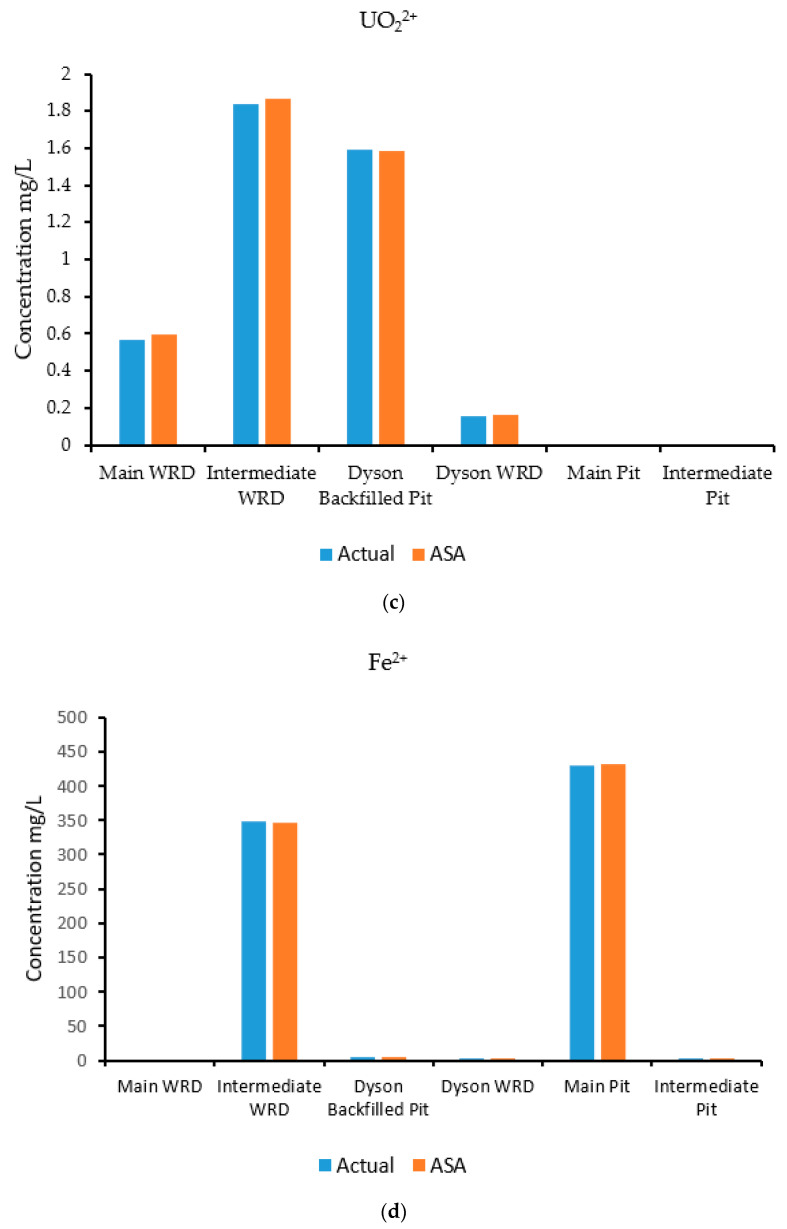
(**a**) Comparison of Cu^2+^ species concentrations (mg/L) estimated by the ASA-linked optimization model and actual data. (**b**) Comparison of SO_4_^2^ species concentrations (mg/L) from the ASA-linked optimization model and synthetic data. (**c**) Comparison of UO_2_^2+^ species concentrations (mg/L) from the ASA-linked optimization model and actual data. (**d**) Comparison of Fe^2+^ species concentrations (mg/L) from the ASA-linked optimization model and the actual data.

This section focuses on a contaminated aquifer site (Figure 7) with six distributed sources of contamination consisting of four waste rock dumps and two open mine pits filled with water. The boundary conditions for flow are represented by the red border lines shown in Figure 8. This case study involves a calibrated model of a real mine site in the Northern Territory, Australia. It is adapted in this study to test the performance of the multiple species source identification model, where individual contaminant species from distributed sources are examined. There were six possible sources: four waste rock dump sites, D1 (Main WRD), D2 (Intermediate WRD), D3 (Dyson Backfilled Pits), D4 (Dyson WRD) and two open pits, P1 (Main Pit) and P2 (Intermediate Pit; Figure 7. There were nine observation wells (W1, W2, W3, W4, W5, W6, W7, W8 and W9) at the study site (Figure 7), which recorded concentrations of six contaminant species over a two-year period. The thickness of the aquifer is 150 m. The aquifer is presumed to be anisotropic. The effective porosity is taken to be 0.3. Table 6 shows the schedule of release of contaminant species, with locations and magnitudes (contaminant concentrations). The releases were assumed to occur over a two-year period and to be continuous thereafter.

A groundwater flow and transport model was run in HYDROGEOCHEM 5.0 to simulate the flow and transport processes and generate synthetic concentrations at specified observation wells. The simulation was carried out for a two-year period with a time-step of 30 days. The concentrations at the selected observation wells estimated by the simulation are shown in Figure 8a–d, which compares actual and predicted concentrations for species at specific observation wells. These concentrations estimated by the simulation model were treated as input observed concentration data for the source identification model. The observed concentration data were fed to the source identification model for characterization of the sources. The optimization algorithm used for this problem was ASA, which tries to find a set of candidate source concentrations of individual contaminants by minimizing the objective function [42,43] defined in Equation (11).

Results and discussion: case studies for model demonstration.

This section discusses the evaluation results obtained from the source identification model using a calibrated model with simulated measurements for testing (as sources are known) the performance. The source identification model was able to identify separate contaminants concentrations. Six sources were used in the characterization. It is important to note that, for this study, the contaminant sources (waste rock dumps) were characterized in terms of concentrations rather than fluxes. This is so because species concentrations are expressed in terms of moles per litre in the software used; hence, concentration multiplied by volume flux gives the mass flux. Additionally, in the case of pits, it was assigned as a boundary condition and hence expressed in terms of concentration.

The results of the source characterization evaluation are presented in Figure 8a–d. Table 7 summarizes the error statistics related to the source characterization using error-free concentration measurement data. Figure 8a–d compare the actual concentrations with those estimated by the optimization model of specific contaminant species at the six sources.

Figure 8a is a graph showing the concentration of the contaminant copper, where the source concentrations are compared with the concentration solutions obtained from the optimization model. The concentrations compared very well at all source locations when error-free data were utilized. Figure 8b shows sulfate concentrations, where the source concentrations are compared with the estimates of the optimization model. The concentrations compared very well at all source locations when error-free data were utilized as synthetic concentration measurements.

Figure 8c shows the concentrations of the contaminant uranium (in the form of UO_2_^2+^), where the source concentrations are compared with the concentration solutions obtained from the optimization model. The actual and estimated concentrations compared very well at all source locations when error-free data were utilized. The Main Pit and Intermediate Pit sources did not show any bars since the source concentration of uranium was near zero at these points. Hence, the optimization model was able to provide same value. Figure 8d shows the concentrations of the contaminant iron (in the form of Fe^2^), where the source concentrations are compared with the concentration solutions obtained from the optimization model. The concentrations compared very well at all locations when error-free synthetic data were used. Source locations at the Main WRD did not peak up, which reflects the same results for the optimization model. At the Intermediate Pit, the concentration of iron was low, so does not show on the figure.

The performance of the linked simulation-optimization source characterization model using an ASA optimization algorithm was evaluated in terms of percent average contaminant source estimation error (PAEE) [44]. PAEE was used to compare the input concentrations of the generated and actual source locations. Mahar and Datta (2001) performed such a comparison in terms of PAEE; using the formula:

(15) $$\mathrm{PAEE}\;(\%)=\frac{\left|{\bar{C}}_{o}-{\tilde{C}}_{o}\right|}{{\tilde{C}}_{o}}\times 100$$

where ${\bar{C}}_{o}$ is the actual input observed species concentration and ${\tilde{C}}_{o}$ is the species concentration generated by the optimization model. 

Using measured concentration data in the optimal source identification model, the actual and optimization-generated species concentrations were accurately estimated within a 10% average estimation error value. This is except for iron, which had a PAEE value of 6.5% at the Intermediate WRD. It can be seen that the PAEE value tends toward zero when the generated concentrations approach the actual ones. Table 7 compares the calculated PAEE values for the species at different source locations.

### 3.3. Source Identification for a Field Problem

This case study considers the transport of reactive contaminant species through an aquifer system. The contamination came from acid mine drainage from waste rock dumps and open mine pits that occurred continuously over a long time. It is associated with an abandoned uranium mine in Australia. The modelled area is approximately 12 km^2^. It is bounded by the upper east branch of the Finniss River on the left side and a specified transient head boundary condition to the borders of the Main and Intermediate Open Pits. The water levels in the open pits were used as the constant head values at the boundaries of the pit. Groundwater contaminant concentrations measured at several monitoring locations were used as specified concentrations in the reactive transport model. These concentrations were estimated as constant values for species at selected monitoring points at specific time intervals. There were six potential sources, consisting of two open pits and four waste rock dumps: OP1, OP2, MWRD, IWRD, DBP and DWRD. These released reactive species contaminants associated with AMD via a reactive transport process over a period of two years. It is assumed that after two years of release, the contaminants stopped being released at the study site. Nine observation wells, labelled OB1, OB2, OB3, OB4, OB5, OB6, OB7, OB8 and OB9, recorded contaminant concentrations over a two-year simulation period.

#### 3.3.1. Results and Discussion of Source Identification for a Field Problem

Based on the concentrations measured at the monitoring wells, source identification was attempted using the optimization formulation in Equation (11). This equation provided optimal source concentrations that matched the field measurements. Corresponding measured concentrations were used to formulate the optimization model used in this study. The concentrations of the species considered were used for optimal source characterization of the distributed sources [44,45,46,47]. 

The optimal source characteristics of the species concentrations obtained from the optimization model were then utilized as input source concentration parameters to model and simulate the concentration plumes from the sources in this study area. 

**Figure 9:**
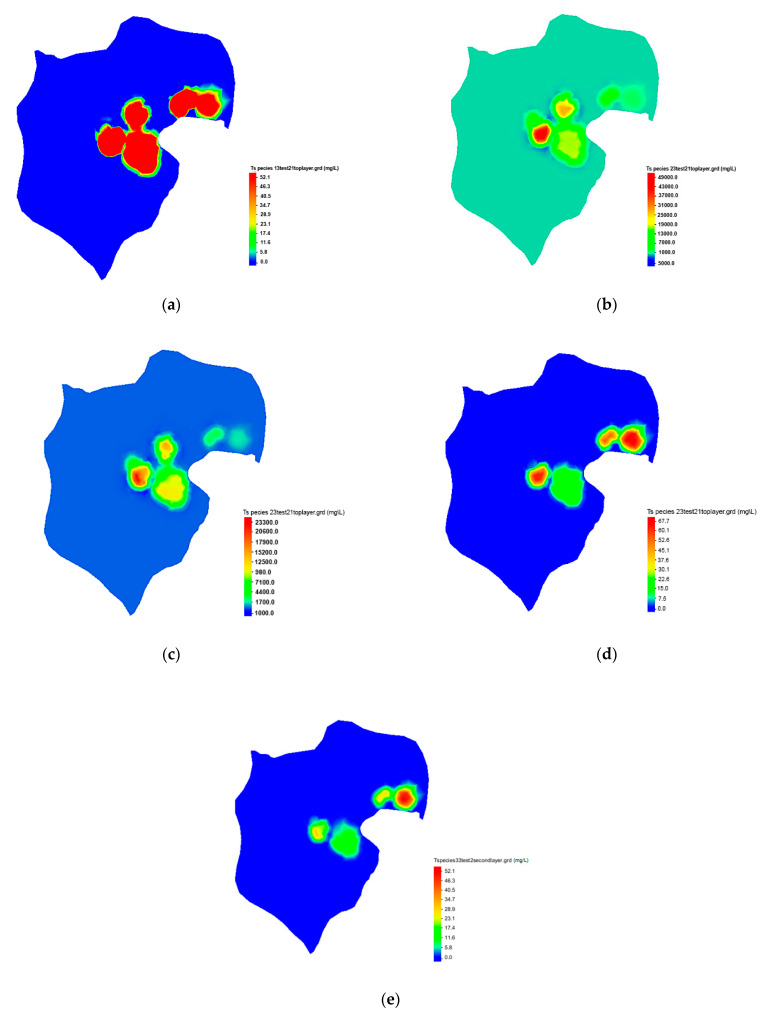
(**a**) Concentration plumes resulting from characterized sources for Species 1: Cu Layer 1. (**b**) Concentration plumes resulting from characterized sources for Species 2: SO_4_ Layer 1. (**c**) Concentration plumes resulting from characterized sources for Species 2: SO_4_ Layer 2. (**d**) Concentration plumes resulting from characterized sources for Species 3: UO_2_ Layer 1. (**e**) Concentration plumes resulting from characterized sources for Species 3: UO_2_ Layer 2.

The use of concentrations measured at monitoring locations to identify sources is discussed. The contours of simulated plumes of different species at different source locations are shown in Figure 9a–e.

Figure 9a shows a simulated contour map of Cu concentration plumes based on field-measured concentrations. Figure 9b,c are simulated contour maps of SO_4_^2+^ concentration plumes represented in Layers 1 and 2, respectively. Comparing Figure 9b,c, it can be seen that as the SO_4_^2+^ moves from one layer to the other, the concentrations at the sources also change. These contour maps show variations in concentrations as the plumes move through different layers of the model. Figure 9d,e show simulated contour maps of UO_2_^2+^ concentration plumes as characterized in Layers 1 and 2, respectively. These contour maps show some variation in the concentrations as the plumes move through different layers of the model. 

Figure 9a–e represent the concentration plumes of species at different layer resulting from characterized sources.

These contours can be used to compare point measurements of concentrations with concentration estimates based on the contours. However, such comparisons are possible only when the actual sources in the field are the same as those identified by the source characterization inverse model, which is not possible in this case. However, few pointwise concentration values seem to be of the same order as those in the field. This may help in an intuitive validation of the source characterization process, as the physical conditions assumed for the performance evaluations resemble the field conditions to a certain extent. Selected points in specific areas in the point concentration comparison and the source identification intuitive validation are discussed below.

The following monitoring bores were used for point concentration comparisons. Two monitoring bores, MB 10-10 and MB 10-11, were positioned below the former copper extraction pad. Monitoring bores MB 10-3 and MB 10-4 were located near the East Finnish Diversion Channel. Monitoring bores PMB 10-5 and PMB 10-6 were situated near the intermediate waste rock dump (IWRD). Monitoring bores MB 10-7, MB 10-12, MB 10-13 and MB 10-16 were located in the central mining area. Monitoring bores PMB 10-9S and PMB 10-9D were sited near the east branch of the Finnish River and the intermediate pit. Monitoring bores PMB 10-20 and PMB 10-21 were also positioned downstream of the mine site. The simulated (sim) and field-observed (obs) concentrations of sulfate, copper, uranium and iron at the monitoring bores are compared in Table 8.

#### 3.3.2. Results for Erroneous Concentration Measurement Values

**Figure 10:**
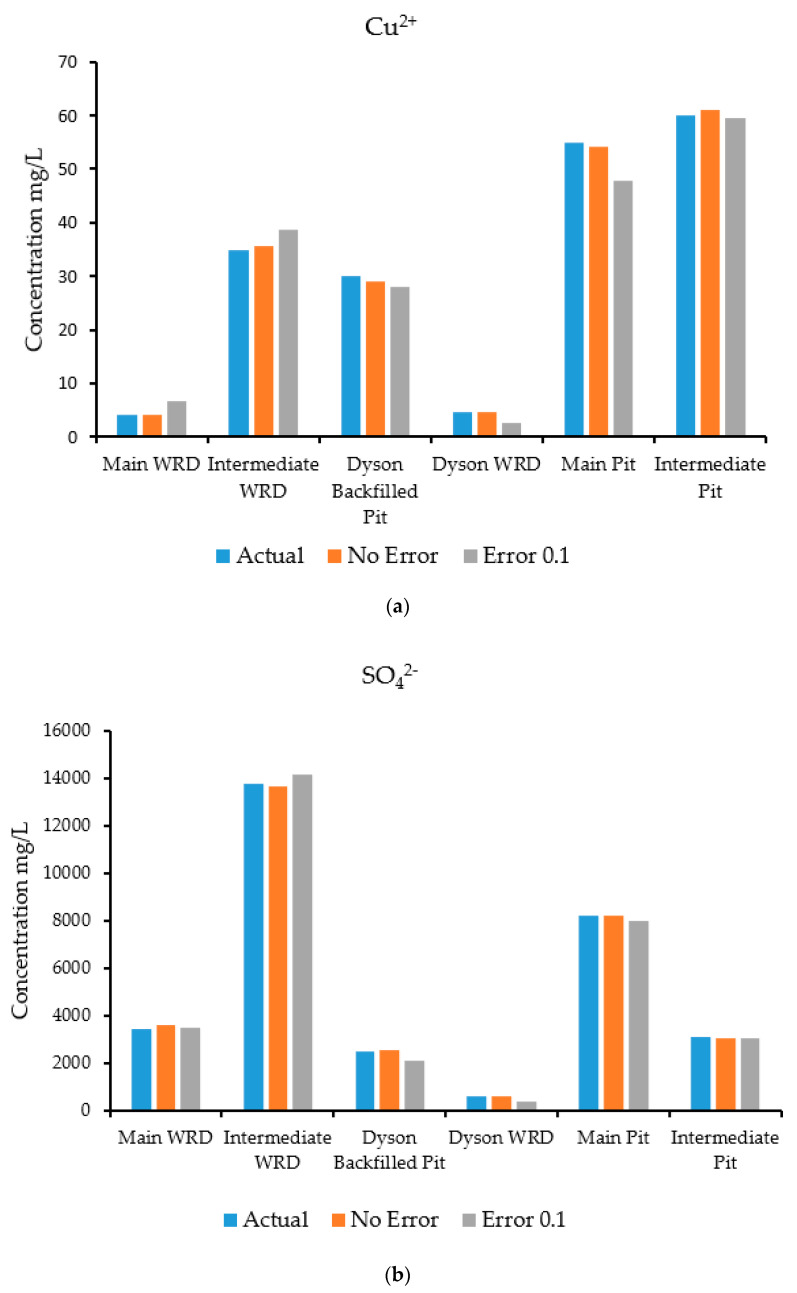

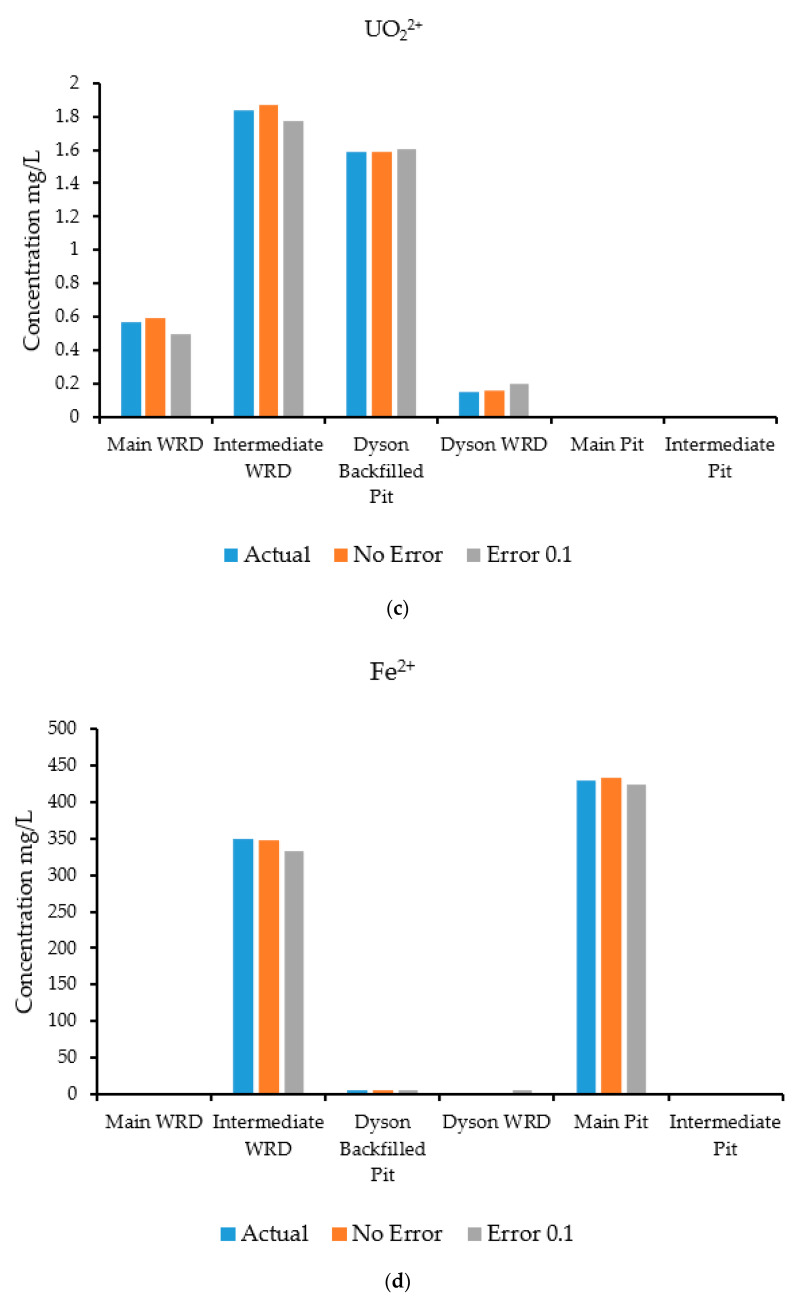
Comparison of species concentrations at sources with a perturbed error of 0.1: (**a**) Cu^2+^ species; (**b**) SO_4_^2-^ species; (**c**) UO_2_^2+^; (**d**) Fe^2+^ species.

**Figure 11:**
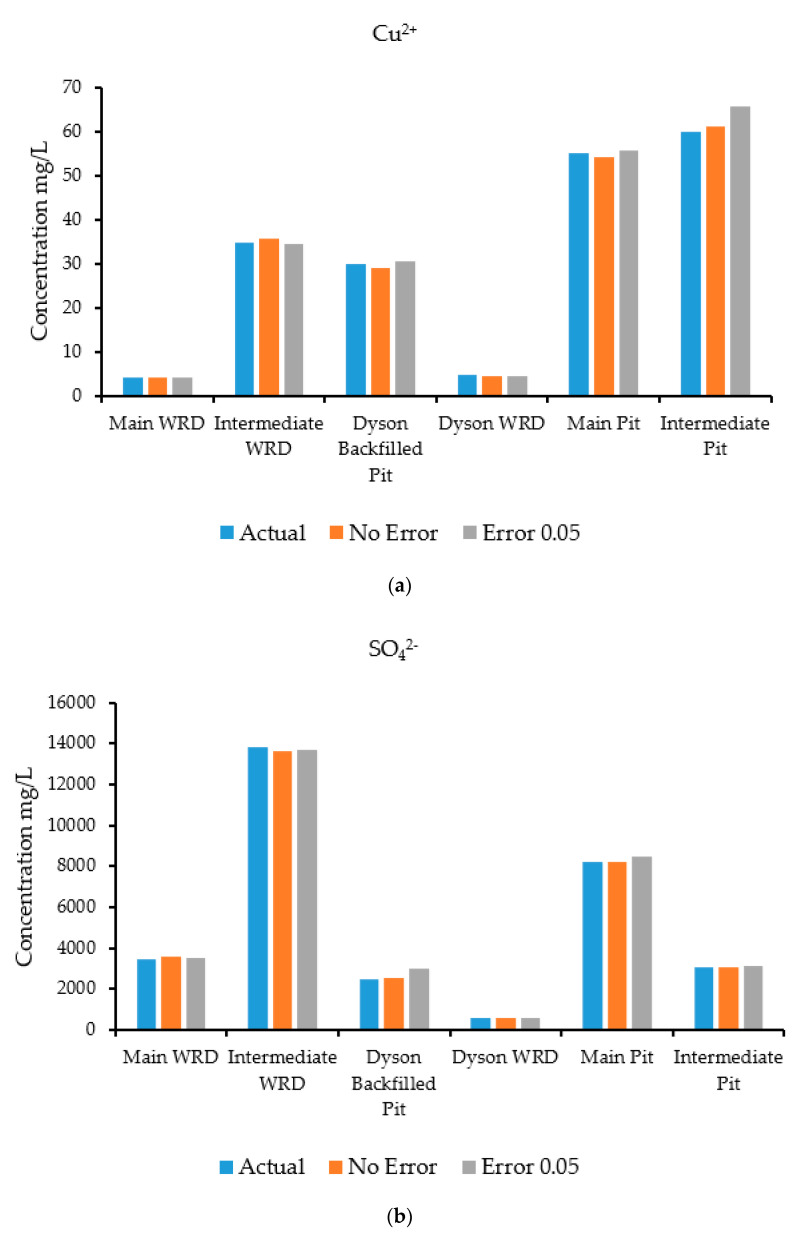

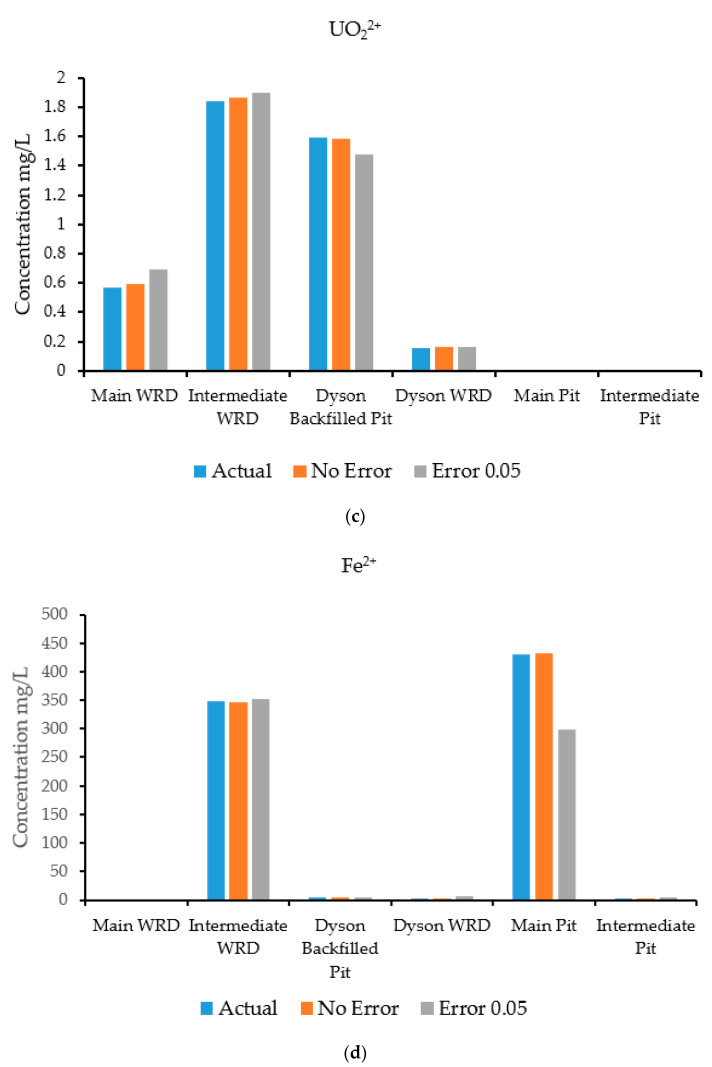
Comparison of species concentrations at sources with a perturbed error of 0.05: (**a**) Cu^2+^ species; (**b**) SO_4_^2+^ species; (**c**) UO_2_^2+^; (**d**) Fe^2+^ species.

**Figure 12:**
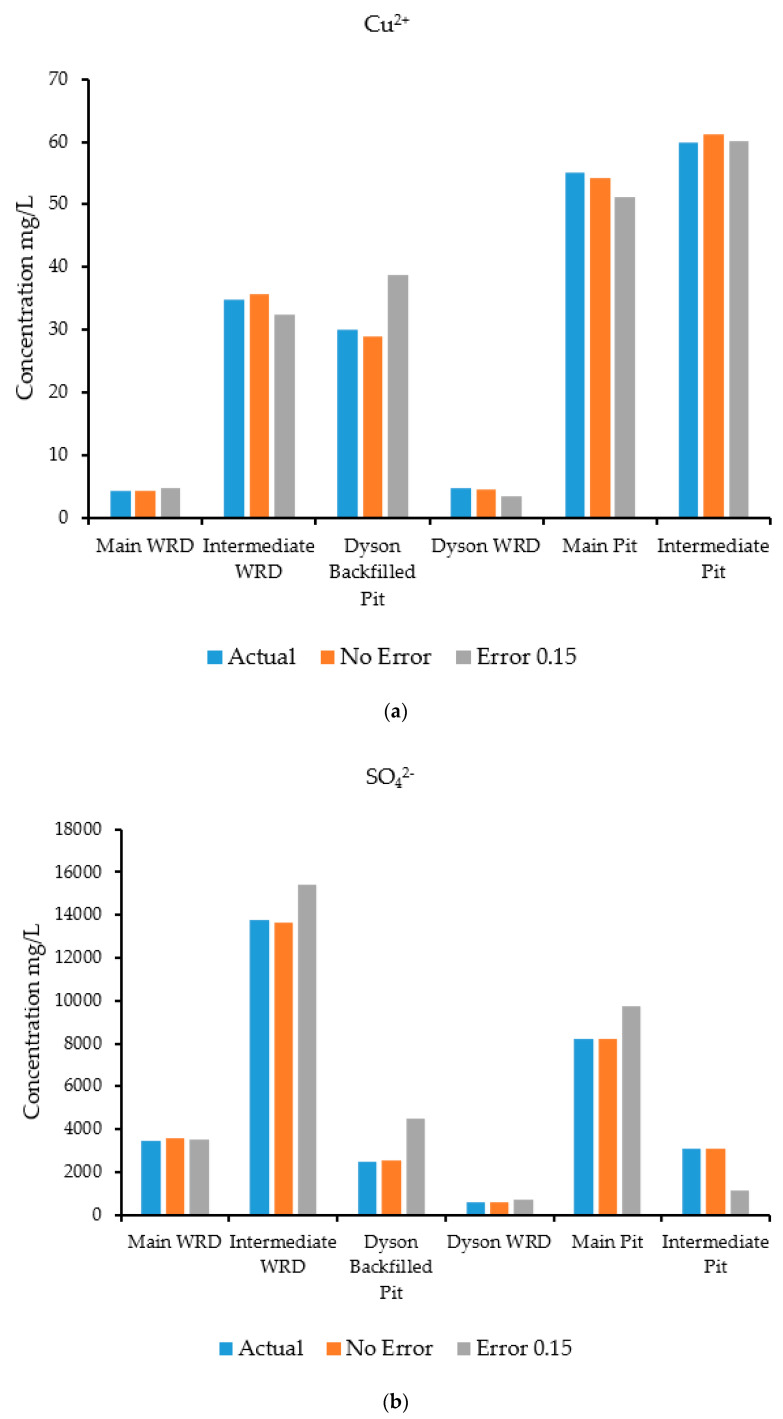

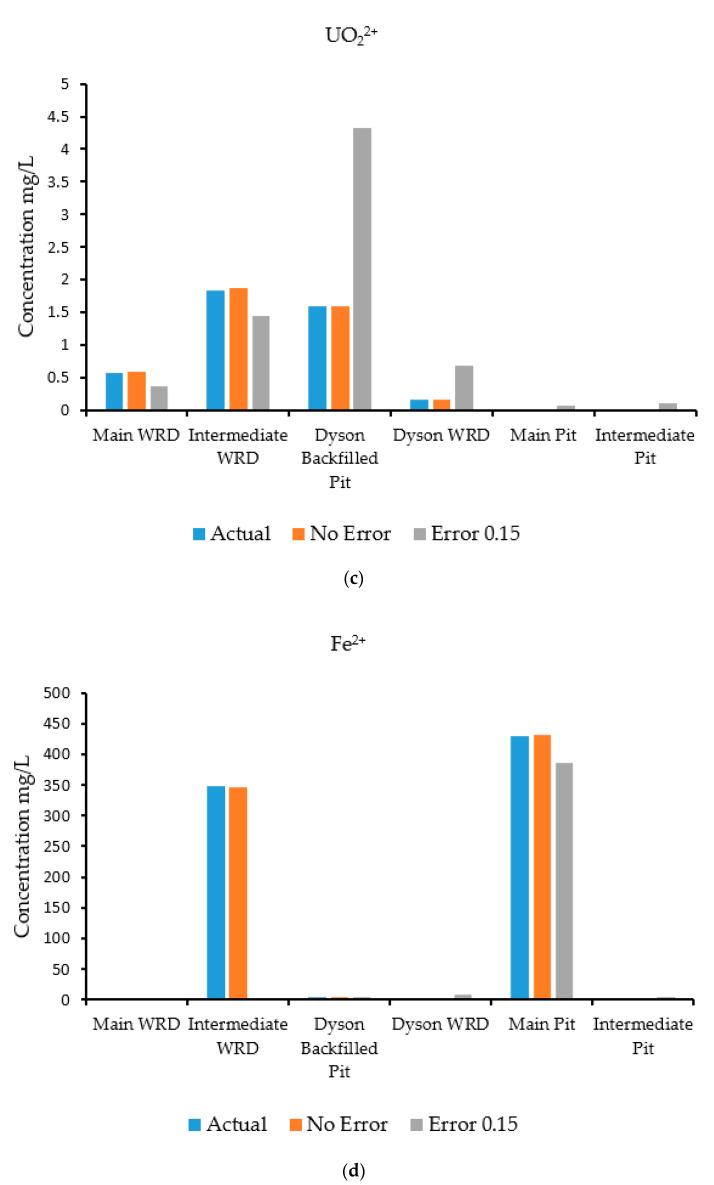
Comparison of species concentrations at sources with a perturbed error of 0.15: (**a**) Cu^2+^ species; (**b**) SO_4_^2-^ species; (**c**) UO_2_^2+^; (**d**) Fe^2+^ species.

**Figure 13:**
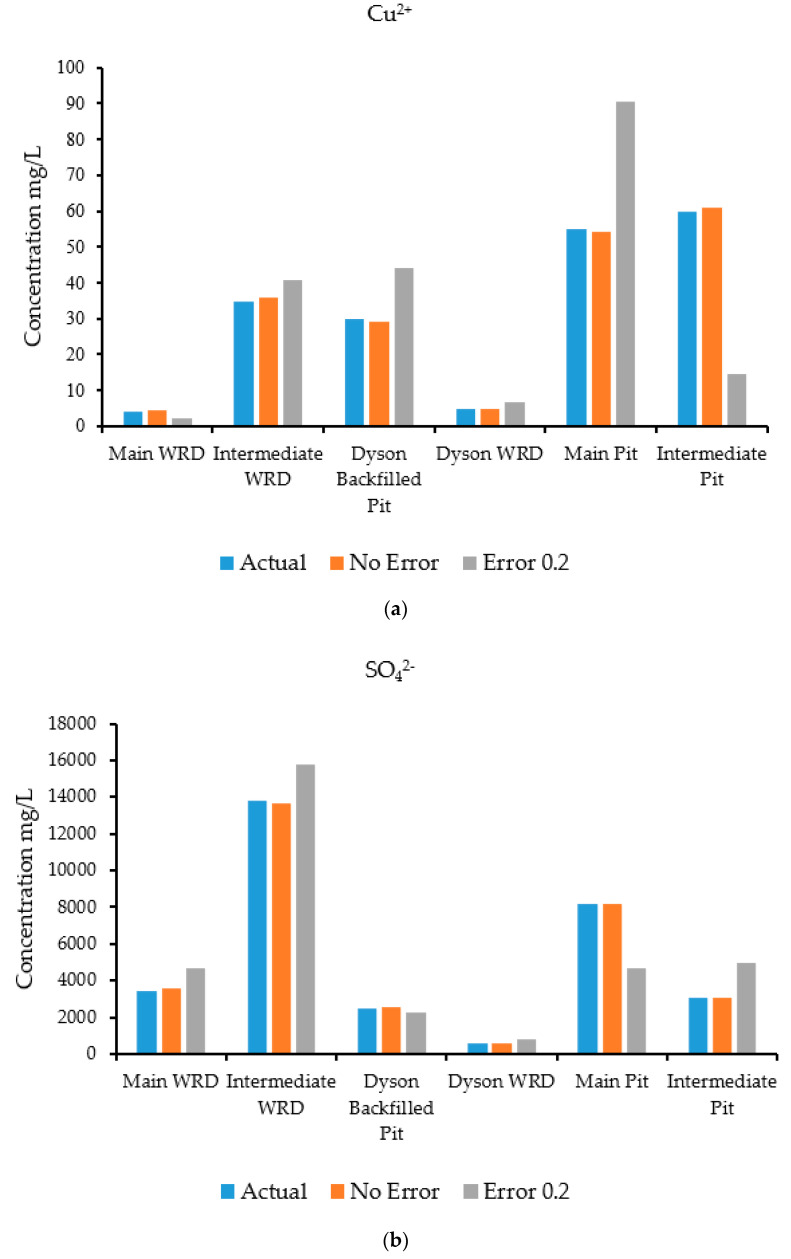

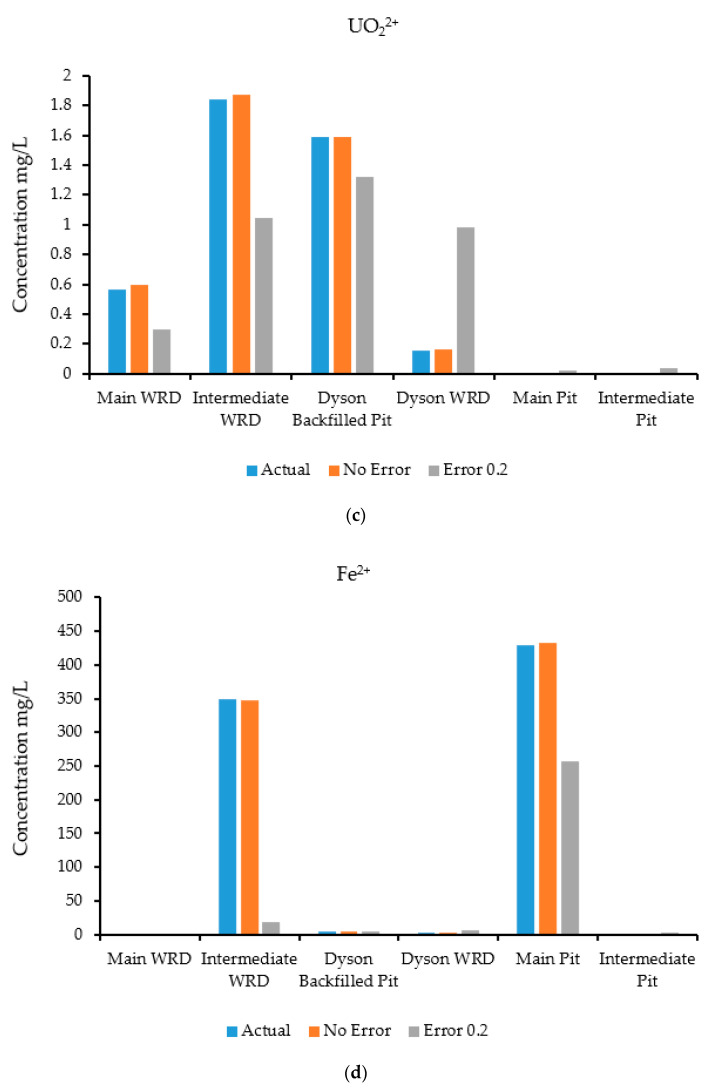
Comparison of species concentrations at sources with a perturbed error of 0.2: (**a**) Cu^2+^ species; (**b**) SO_4_^2-^ species; (**c**) UO_2_^2+^; (**d**) Fe^2+^ species.

Figure 10, Figure 11, Figure 12 and Figure 13 compare the ASA-generated species concentrations at the distributed sources with error perturbation values of 0.05, 0.10, 0.15 and 0.2. Each of the unknown species’ source concentrations is marked on the *y* axes. The *x* axes have three bars for each potential source location corresponding to concentration of a species at the source. The first bar is the actual value, the second represents estimated values based on error-free concentration measurements, and the third bar represents estimated values based on concentration measurements with perturbed erroneous measurement data.

The results of source concentration identification using error-free data closely match those using the actual source concentration values for all species and source areas, as displayed in Figure 10, Figure 11, Figure 12 and Figure 13.

**Figure 14:**
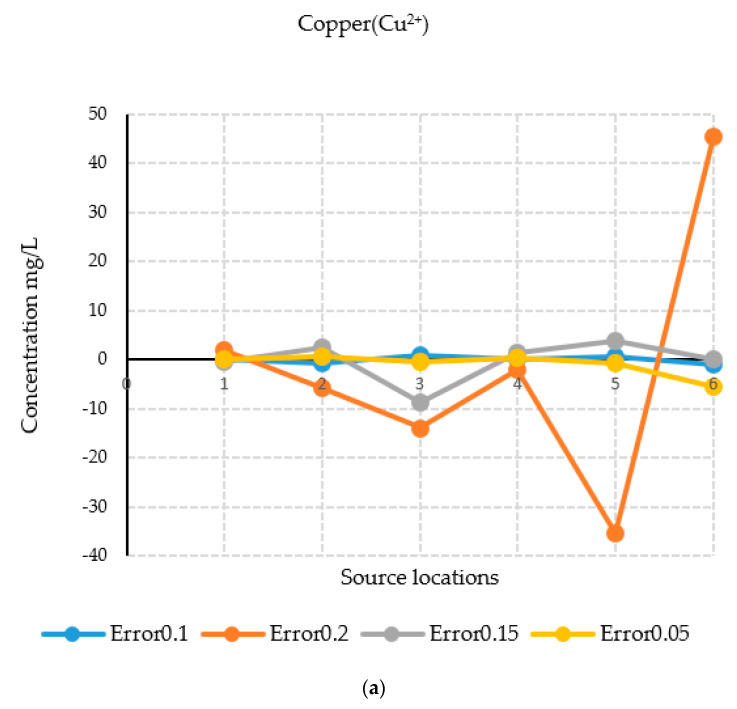

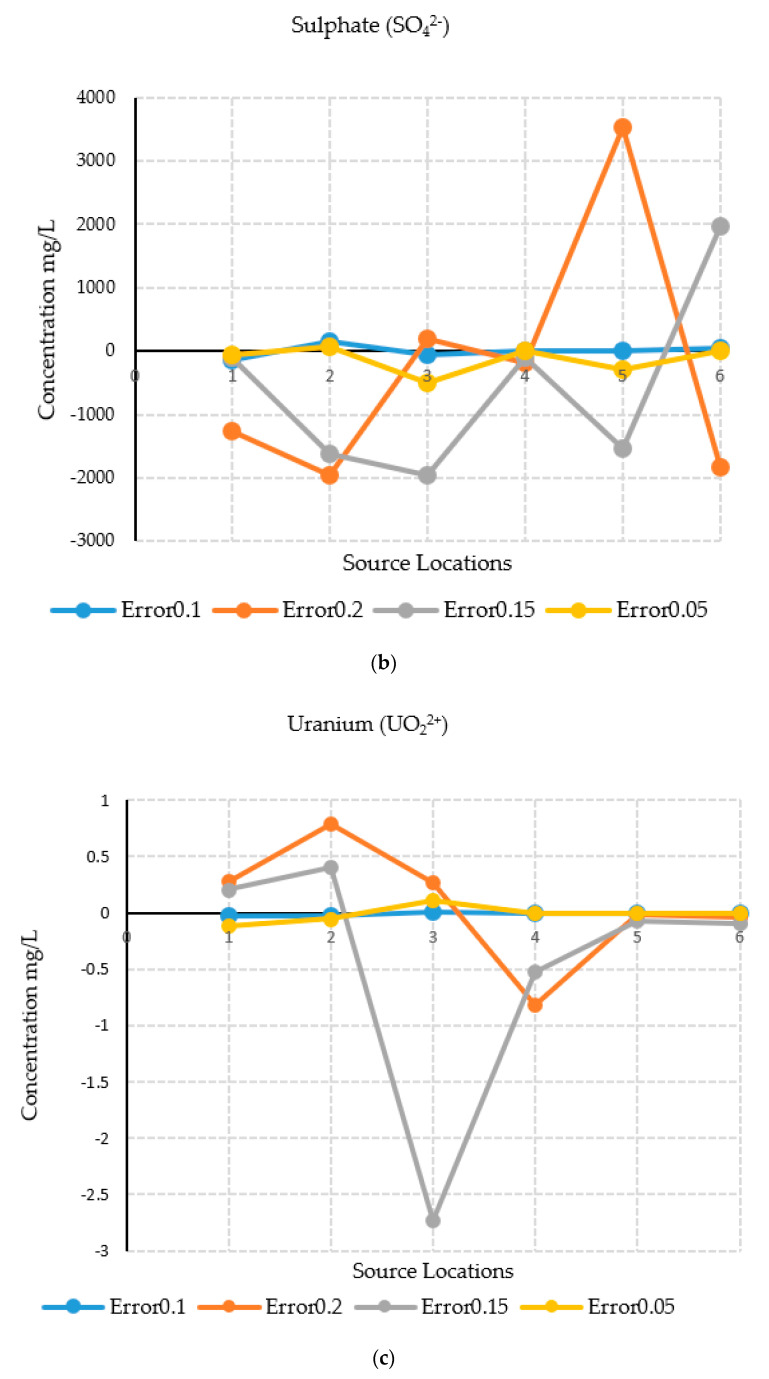

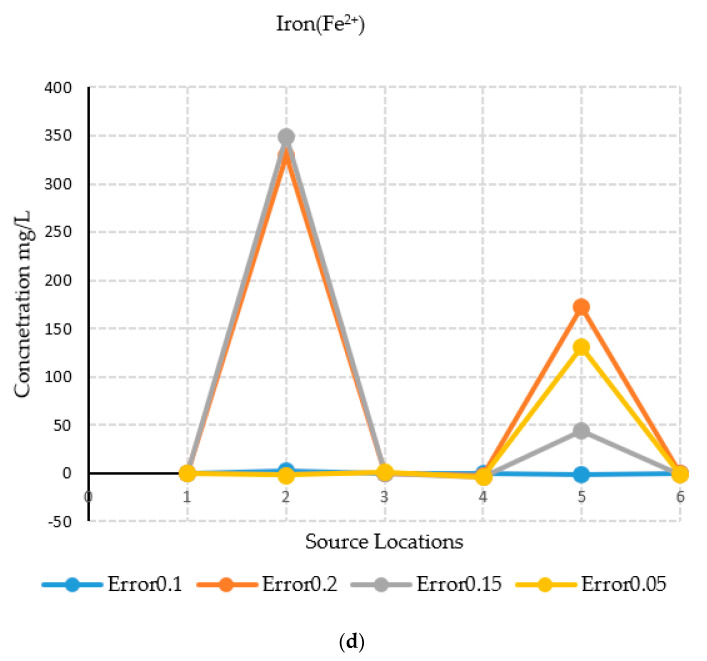
Comparison of the source identification errors obtained using different erroneous measurement data: (**a**) Cu^2+^ species; (**b**) SO_4_^2-^ species; (**c**) UO_2_^2+^ species; (**d**) Fe^2+^ species.

Figure 14a–d show the errors in source estimation for different scenarios. The figures compare the errors in a linear format with four error perturbation values of 0.1, 0.2, 0.15 and 0.05. The error graph justifies the fact that when concentrations are perturbed, as in the case of field measurements where uncertainties in measurement or recording are likely, the optimization model shows that it is robust and capable of handling any form of data and, at the same time, providing an optimal solution of source characteristics with a realistic margin of error. Figure 14a–d show how minimal errors are observed when erroneous data (randomly perturbed) are used as input for source characterization. In Figure 14a–d, the *y* axis represents source estimation errors. The *x* axis depicts the different source locations, where 1 = Main WRD, 2 = Intermediate WRD, 3 = Dyson Backfilled, 4 = Dyson WRD, 5 = Main Pit, and 6 = Intermediate Pit.

## 4. Conclusions

This study involved as a first step, the development and calibration of a transient groundwater flow model for a contaminated aquifer underlying an abandoned uranium mine in the Northern Territory of Australia. This work was initiated as a fundamental step to understand the flow of the aquifer system and to establish a multiple species reactive transport model for a hydrogeologically complex contaminated aquifer. The outcome of the model is the basis for a transient flow model and for reactive transport and predictive modelling. The implementation of this model was based on subjective judgment of the selection of appropriate data, due to their sparseness and reliability. The numerical flow model was calibrated a two-year period of 2010–2012. The calibrated model was also validated according to selected hydrogeological parameters with data from 2012 to 2014. The calibrated flow model and transport model were used to simulate the heads and concentrations at various points in the study area. Overall, the calibrated model provided a reasonable match with field observations, demonstrating strong hydraulic connection to the materials at each layer. This validation process suggests that the current calibrated model is a reasonable approximation of the current flow conditions at the Rum Jungle Mine site and can be used to predict the responses of the groundwater system. 

The challenge in using reactive transport models for modelling real-life scenarios is in selecting the geochemical reactions that best describe the geochemical processes occurring at the study site and transforming them into transport equations of (1) kinetic variables, (2) components and (3) equilibrium variables. These can then be used for the computation of equilibrium and kinetic rates to facilitate numerical contaminant transport simulations.

There was an acceptable level of agreement between the observed and simulated hydraulic heads. Transient simulation of the groundwater hydraulic heads and movement of reactive contaminants was accomplished with the developed simulation model. The concentrations predicted for the species in the reaction network are of the same order of magnitude as those of available measurements. Quantification of the contaminant sources in terms of magnitude, location and duration of activity was not possible. Therefore, the contaminant transport simulation calibration is, at best, subjective in this scenario. Hence, the simulation models, once reasonably calibrated to site conditions, are potentially good approximations, and similar approaches can be used for similar sites with similar complex challenges.

The main aim of this study was to develop and demonstrate the utility of a source characterization methodology applied to a mining site-contaminated aquifer that is no longer used. The method uses a linked ASA optimization algorithm to model the transport of multiple chemically-reactive species in a contaminated aquifer beneath a former mine site in Australia. A numerical simulation model of the flow and transport processes was developed that incorporates heterogeneous, anisotropic, hydrogeological parameters. A performance evaluation demonstrated the potential applicability of this method to simultaneously identifying the spatial distributions and input concentrations of unknown areal groundwater contaminant sources. The method uses a limited amount of contaminant species concentration data at different time intervals and monitoring well locations. 

The preliminary evaluation results are encouraging and point towards the feasibility of using the proposed method to optimally characterize the sources and pathways of contamination in complex aquifers. The range of errors obtained for error and erroneous cases ranges between 4% and 16%, etc. 

This proposed methodology can overcome some of the shortcomings of some of the currently available methods applied to optimal characterization of unknown contaminant sources, particularly at very complex contaminated aquifer sites such as abandoned mines that contain multiple species of reactive chemical contaminants. Such characterization is an essential initial step for solving critical environmental problems and designing effective contamination remediation strategies. This study also highlights limitations in utilizing and implementing calibrated flow and transport simulation models calibrated with very limited available site measurements, particularly in hydrogeologically and geochemically complex contaminated aquifer sites. The issue of efficient and adequate monitoring network design for improving the reliability and accuracy of unknown source identification remains a challenge and needs further attention [48,49].

## Figures and Tables

**Table 1:** The study aquifer’s physical and hydrogeological properties.

Parameter	Value
Number of nodes	6587
Number of elements	10,704
Effective porosity, θ	0.28
Longitudinal dispersivity, αL	10 m/d
Transverse dispersivity, αT	0.1 m/d
Vertical dispersivity, αV	0.01
Average rainfall	2372 mm/year
Number of nodes	6587

**Table 2:** Hydraulic conductivity and layer thickness values of the study aquifer.

Model Layer	Hydraulic Conductivity		Thickness (m)
	K_x_ (m/day)	K_z_ (m/day)	
Layer 1	0.13	0.12	3
Layer 2	1.21	1.21	7.5
Layer 3	0.44	0.44	7.5
Layer 4	0.65	0.65	30
Layer 5	0.11	0.11	60
Layer 6	0.04	0.04	45

**Table 3:** Typical chemical reactions during contaminant transport.

Chemical Reaction	Constant Rate (log K)
H_2_O(aq) = H^+^ + OH^−^	−13.99
H^+^ + SO_4_^2−^ = HSO_4_^-^	1.99
Cu^2+^ + H_2_O = Cu(OH)^+^ + H^+^	−9.19
Cu^2+^ + SO_4_^2-^ = CuSO_4_	2.36
Cu^2+^ + 2H_2_O = Cu(OH)_2_ + 2H^+^	−16.19
Cu^2+^ + 3H_2_O = Cu(OH)_3_^-^ + 3H^+^	−26.9
Fe^2+^ + H_2_O = H^+^ + FeOH^+^	−9.50
Fe^2+^ + SO_4_^2-^ = FeSO_4_	2.20
Fe^2+^ + 2H_2_O = 2H^+^ + Fe(OH)_2_	−20.57
Fe^2+^ + 3H_2_O = 3H^+^ + Fe(OH)_3_^−^	−31.00
Fe^2+^ + 4H_2_O = 4H^+^ + Fe(OH)_4_^2−^	−46.00
Fe^2+^ + 2H_2_O = 2H+ Fe(OH)_2_	−12.10
Mn^2+^ + SO_4_^2−^ = MnSO_4_	2.26
Mn^2+^ + H_2_O = MnOH^+^ + H^+^	−10.59
Mn^2+^ + 3H_2_O = Mn(OH)_3_^−^ + 3H^+^	−34.08
UO_2_^2+^ + SO_4_^2−^ = UO_2_SO_4_	2.95
UO_2_^2+^ + SO_4_^2-^ = UO_2_(SO4)_2_^2−^	4.00
2UO_2_^2+^ + 2H_2_O = (UO_2_)_2_(OH)_2_^2+^ + 2H^+^	−5.68
UO_2_^2+^ + 2H_2_O = 2H^+^ + (UO_2_)(OH)	−5.40
Fe(OH)_3(s)_ +3H^+^ = Fe^3+^ + 3H_2_O	Kf = 0.05
FeOOH_(S)_ + 3H^+^ = Fe^3+^ + 3H_2_O	Kf = 0.07
FeOOH_(S)_ = FeOH	Kf = 0.05

All equations specified in Table 3 were derived by modifying equations based on [16].

**Table 4:** Validation of hydraulic head values for 2015 (values in m AHD).

Monitoring Well	Observed Head (m)	Simulated Head (m)	Residual (m)
PMB1a	63.42	63.01	0.41
PMB1b	63.86	62.91	0.95
PMB4	62.44	63.03	−0.59
PMB5	58.26	59.13	−0.87
PMB6	58.76	58.59	0.17
PMB7	58.55	58.48	0.07
PMB8D	59.48	59.24	0.24
PMB10	59.62	59.81	−0.19
PMB11	59.14	59.66	−0.52
PMB12	59.76	60.04	−0.28
PMB13	60.21	60.04	0.17
PMB14	61.35	60.47	0.88
PMB17	60.65	60.02	0.63
PMB23	58.76	59.16	−0.4
RN22039	61.57	60.8	0.77
RN22081	60.98	61.23	−0.25
RN22085	65.12	64.49	0.63
RN22543	58.5	59.18	−0.68
RN23051	63.16	63.55	−0.39
RN23413	63.42	62.96	0.46
RN23419	63.37	63.59	−0.22
RN29993	62.88	63.36	−0.48

**Table 5:** Statistics of the residuals for the calibration and validation periods.

Statistic	Calibrated Period	Validation Period
Simulation period (days)	730	730
MAE (m)	0.82	0.72
Standard deviation (m)	0.77	0.91
NRMSE (m)	0.09	0.10
RMSE (m)	1.10	0.91

**Table 6:** Contamination release schedule from distributed sources—simulated measurements for testing (as sources are known).

Site	Contaminant Concentration (mg/L)			
	Cu^2+^	SO_4_^2-^	UO_2_^2+^	Fe^2+^
Main WRD	4.19	3430	0.568	0
Intermediate WRD	34.9	13800	1.840	349
Dyson Backfilled Pits	30.0	2500	1.590	4.865
Dyson WRD	4.63	579	0.155	2.74
Main Pit	55.0	8200	0.000	430
Intermediate Pit	60.0	3100	0.000	2.00

**Table 7:** Comparison of percent average estimation error (PAEE, %) obtained for species at distributed source locations using error-free data.

Source	PAEE (%)			
	Cu	SO_4_^2−^	U	Fe
Main WRD	0.1	3.8	4.3	0.0
Intermediate WRD	2.3	1.10	1.55	6.5
Dyson Backfilled Pit	3.32	2.35	0.26	5.35
Dyson WRD	0.33	1.99	5.5	5.22
Main Pit	1.30	0.11	0	0.50
Intermediate Pit	1.82	1.35	0	4.0

**Table 8:** Comparison of observed (Obs) and simulated (Sim) contaminant concentrations (mg/L) at monitoring locations based on final optimal sources from the source characterization model.

	Sulphate (SO_4_^2+^)		Iron (Fe^2+^)		Copper (Cu^2+^)		Magnesium (Mn^2+^)		Uranium (UO_2_^2+^)	
Bore	Obs	Sim	Obs	Sim	Obs	Sim	Obs	Sim	Obs	Sim
PMB 10-3	493	500	0.542	0.55	2.41	3.00	1.81	1.79	0.073	0.07134
PMB 10-4	1250	1511	0.336	0.4	0.015	0.019	0.117	0.115	0.012	0.009117
PMB 10-5	212	200	0.054	0.06	0.001	0	0.084	0.09	0.003	0
PMB 10-6	1090	1050	0.03	0.04	0	0	0.649	0.7127	0.002	0
PMB 10-10	756	650	1.8	1.6	0.006	0.001112	0.22	0.21	0.085	0.0964
PMB 10-11	5180	5170	1.49	1.55	77.2	80	144	150	0.024	0.035
PMB 10-22	3810	3800	37.4	37.1	561	590	124	140	0.109	0.1763
PMB 10-24	1050	1000	0.56	0.61	52.6	55	18.3	20	0.162	0.113
RN022543	1340	1200	0	0	0.008	0.009	0.081	0.089	0.003	0.00354
RN022543	1140	1020	0.014	0.017	0.018	0.015	0.069	0.075	0.003	0.003
PMB 10-7	1450	1400	0.002	0.002113	0.003	0.002	0.002	0.01	0.006	0.005126
PMB 10-9S	350	285	0.22	0.33	0.003	0.002	0.474	0.5	0.009	0.008
PMB 10-9D	3270	3300	2.35	2.4	0.040	0.03	5.42	5.66	0.316	0.611

## References

[1] Datta B., Kourakos G. (2015). Preface: Optimization for groundwater characterization and management. Hydrogeol. J..

[2] Atmadja J., Bagtzoglou A. (2001). State of the art report on mathematical methods for groundwater pollution source identification. Environ. Forensics.

[3] Amirabdollahian M., Datta B. (2013). Identification of contaminant source characteristics and monitoring network design in groundwater aquifers: An overview. J. Environ. Prot..

[4] Atmadja J., Bagtzoglou A.C. (2001). Pollution source identification in heterogeneous porous media. Water Resour Res..

[5] Ayvaz M.T. (2010). A linked simulation-optimization model for solving the unknown groundwater pollution source identification problems. J. Contam. Hydrol..

[6] Ayvaz M.T. A new simulation-optimization approach for simultaneously identifying the spatial distribution and source fluxes of the areal groundwater pollution sources. Proceedings of the E-Proceedings of the 36th IAHR World Congress.

[7] Ayaz M. (2017). Groundwater pollution source identification using genetic algorithm based optimization model. Int. J. Comput. Sci. Eng..

[8] Chadalavada S., Datta B., Naidu R. (2011). Optimisation approach for pollution source identification in groundwater: An overview. Int. J. Environ. Waste Manag..

[9] Hayford M., Datta B. (2018). Geochemical reactive modeling of flow and transport process at a mine site in Northern Territory, Australia. Int. J. Geomate.

[10] Hayford M. (2020). Development and Evaluation of Models for Assessing Geochemical Pollution Sources with Multiple Reactive Chemical Species for Sustainable Use of Aquifer Systems: Source Characterization and Monitoring Network Design. Ph.D. Thesis.

[11] Chaddalavada S., Datta B., Naidu R. (2012). Optimal identification of groundwater pollution sources using feedback monitoring information: A case study. Environ. Forensics.

[12] Datta B., Chakrabarty D., Dhar A. (2009). Simultaneous identification of unknown groundwater pollution sources and estimation of aquifer parameters. J. Hydrol..

[13] Datta B., Petit C., Palliser M., Esfahani H.K., Prakash O. (2017). Linking a simulated annealing based optimization model with PHT3D simulation model for chemically reactive transport processes to optimally characterize unknown contaminant sources in a former mine site in Australia. J. Water Resour. Prot..

[14] Prakash O., Datta B. (2014). Multiobjective monitoring network design for efficient identification of unknown groundwater pollution sources incorporating genetic programming-based monitoring. J. Hydrol. Eng..

[15] Esfahani H.K., Datta B. (2018). Fractal singularity-based multiobjective monitoring networks for reactive species contaminant source characterization. J. Water Resour. Plan. Manag..

[16] Agboola O., Babatunde D., Isaac Fayomi O., Sadiku E., Popoola P., Moropeng L., Yahaya A., Mamudua O.A. (2020). A review on the impact of mining operation: Monitoring, assessment and management. Results Eng..

[17] Jamshidi A., Samani J., Samani H., Zanini A., Tanda M., Mazaheri M. (2020). Solving Inverse Problems of Unknown Contaminant Source in Groundwater-River Integrated Systems Using a Surrogate Transport Model Based Optimization. Water.

[18] Amirabdollahian M., Datta B., Beck P.H. (2019). Application of a link simulation optimization model utilizing quantification of hydrogeologic uncertainty to characterize unknown groundwater contaminant sources. Model. Earth Syst. Environ..

[19] Jha M., Datta B. (2015). Application of unknown groundwater pollution source release history estimation methodology to distributed sources incorporating surface-groundwater interactions. Environ. Forensics.

[20] Ayaz M.T. (2016). A hybrid simulation-optimization approach for solving the areal groundwater pollution source identification problems. J. Hydrol..

[21] Esfahani H.K., Datta B. (2016). Linked optimal reactive contaminant source characterization in contaminated mine sites: Case study. J. Water Resour. Plan. Manag..

[22] Yeh G.T., Sun J.T., Jardine P.M., Burger W.D., Fang Y.L., Li M.H., Siegel M.D. (2004). HYDROGEOCHEM 5.0: A Three-Dimensional Model of Coupled Fluid Flow, Thermal Transport, and Hydrogeochemical Transport through Variably Saturated Conditions—Version 5.0.

[23] Ingber L. (1989). Very fast simulated re-annealing. Math. Comput. Model..

[24] Ingber L. (1993). Simulated annealing: Practice versus theory. Math. Comput. Model..

[25] Ingber L., Rosen B. (1992). Genetic algorithms and very fast simulated reannealing: A comparison. Math. Comput. Model..

[26] Robertson GeoConsultants Inc RGC (2010). Phase 1 Report: Initial Review and Data Gap Analysis.

[27] Robertson GeoConsultants Inc. (2012). Phase 3 (Stage 2 Report)—Groundwater Flow Model for the Rum Jungle Mine.

[28] Fang Y., Yeh G.-T., Burgos W.D. (2003). A general paradigm to model reaction-based biogeochemical processes in batch systems. Water Resour. Res..

[29] Robertson GeoConsultants Inc. (2010). RGC. Technical Specifications for 2010 Rum Jungle Drilling Program.

[30] Robertson GeoConsultants Inc. (2011). RGC. Phase 2 Report: Detailed Water Quality Review and Preliminary Contaminant Load.

[31] Robertson GeoConsultants Inc. (2011). RGC. Phase 3 (Stage 1 Report): Development of Conceptual Flow Model for the Rum Jungle Mine.

[32] Robertson GeoConsultants Inc. (2016). RGC. Phase 3 (Stage 1 Report)—Groundwater Flow and Transport Model for current conditions, Rum Jungle Mine.

[33] Northern Territory Department of Mines and Energy (2003). Conceptual Rehabilitation Plan Report—Former Rum Jungle Mine Site.

[34] Ahmad M., Lally J.H., McCready A.J. (2006). Economic geology of the Rum Jungle Mineral Field, Northern Territory. North. Territ. Geol. Surv..

[35] Gorelick S.M., Evans B., Remson I. (1983). Identifying sources of groundwater pollution: An optimization approach. Water Resour. Res..

[36] Gurarslan G., Karahan H. (2015). Solving inverse problems of groundwater-pollution-source identification using a differential evolution algorithm. Hydrogeol. J..

[37] Singh R.M., Datta B. (2006). Identification of groundwater pollution sources using GA-based linked simulation optimization model. J. Hydrol. Eng..

[38] Prakash O., Datta B. (2014). Characterization of groundwater pollution sources with unknown release time history. J. Water Resour. Prot..

[39] Prakash O., Datta B. (2015). Optimal characterization of pollutant sources in contaminated aquifers by integrating sequential-monitoring-network design and source identification: Methodology and an application in Australia. Hydrogeol. J..

[40] Ferguson P.R., Wels C., Fawcett M. Current Water Quality Conditions at the Historic Rum Jungle Mine Site, Northern Australia. Proceedings of the 9th International Conference on Acid Rock Drainage (ICARD).

[41] Doherty J. (2015). PEST—The Book: Calibration and Uncertainty Analysis for Complex Environmental Models.

[42] Jha M., Datta B. (2013). Three-dimensional groundwater contamination source identification using adaptive simulated annealing. J. Hydrol. Eng..

[43] Mahar P.S., Datta B. (2001). Optimal identification of ground-water pollution sources and parameter estimation. J. Water Resour. Plan. Manag..

[44] Mahar P.S., Datta B. (2000). Identification of pollution sources in transient groundwater systems. Water Resour. Manag..

[45] Sreekanth J., Datta B. (2015). Review: Simulation-optimization models for the management and monitoring of coastal aquifers. Hydrogeol. J..

[46] Jha M., Datta B. (2015). Application of dedicated monitoring-network design for unknown pollutant-source identification based on dynamic time warping. J. Water Resour. Plan. Manag..

[47] Ketabchi H., Ataie-Ashtiani B. (2015). Review: Coastal groundwater optimization—advances, challenges, and practical solutions. Hydrogeol. J..

[48] Mahar P.S., Datta B. (1997). Optimal monitoring network and ground-water-pollution source identification. J. Water Resour. Plan. Manag..

[49] Dhar A., Datta B. (2010). Logic based design of groundwater monitoring network for redundancy reduction. J. Water Resour. Plan. Manag..

